# Epigenetic Regulation of Phenotypic Sexual Plasticity Inducing Skewed Sex Ratio in Zebrafish

**DOI:** 10.3389/fcell.2022.880779

**Published:** 2022-07-15

**Authors:** Shahrbanou Hosseini, Nares Trakooljul, Marc Hirschfeld, Klaus Wimmers, Henner Simianer, Jens Tetens, Ahmad Reza Sharifi, Bertram Brenig

**Affiliations:** ^1^ Molecular Biology of Livestock and Molecular Diagnostics Group, Department of Animal Sciences, University of Goettingen, Göttingen, Germany; ^2^ Functional Breeding Group, Department of Animal Sciences, University of Goettingen, Göttingen, Germany; ^3^ Institute of Veterinary Medicine, University of Goettingen, Göttingen, Germany; ^4^ Center for Integrated Breeding Research (CiBreed), University of Goettingen, Göttingen, Germany; ^5^ Research Institute for Farm Animal Biology (FBN), Institute of Genome Biology, Genomics Unit, Dummerstorf, Germany; ^6^ Animal Breeding and Genetics Group, Department of Animal Sciences, University of Goettingen, Göttingen, Germany

**Keywords:** DNA methylation, RRBS, phenotypic sexual plasticity, zebrafish, sex-biased family, sex ratio variation

## Abstract

The plasticity of sexual phenotype in response to environmental conditions results in biased sex ratios, and their variation has an effect on population dynamics. Epigenetic modifications can modulate sex ratio variation in species, where sex is determined by genetic and environmental factors. However, the role of epigenetic mechanisms underlying skewed sex ratios is far from being clear and is still an object of debate in evolutionary developmental biology. In this study, we used zebrafish as a model animal to investigate the effect of DNA methylation on sex ratio variation in sex-biased families in response to environmental temperature. Two sex-biased families with a significant difference in sex ratio were selected for genome-wide DNA methylation analysis using reduced representation bisulfite sequencing (RRBS). The results showed significant genome-wide methylation differences between male-biased and female-biased families, with a greater number of methylated CpG sites in testes than ovaries. Likewise, pronounced differences between testes and ovaries were identified within both families, where the male-biased family exhibited a higher number of methylated sites than the female-biased family. The effect of temperature showed more methylated positions in the high incubation temperature than the control temperature. We found differential methylation of many reproduction-related genes (e.g., *sox9a, nr5a2, lhx8a, gata4*) and genes involved in epigenetic mechanisms (e.g., *dnmt3bb.1, dimt1l*, *hdac11, h1m*) in both families. We conclude that epigenetic modifications can influence the sex ratio variation in zebrafish families and may generate skewed sex ratios, which could have a negative consequence for population fitness in species with genotype-environment interaction sex-determining system under rapid environmental changes.

## Introduction

In evolutionary developmental biology, the mechanism of sex determination is one of the most important developmental processes in bipotential gonad to differentiate into a testis or an ovary and establish the sex of an organism in a binary fate decision. Gonochoristic vertebrates exhibit two major types of sex determination, namely genotypic sex determination (GSD) and environmental sex determination (ESD). In organisms with GSD, sex is determined at the time of fertilization by inheritance of genetic factors, whereas in organisms with ESD, sex is determined after fertilization in response to environmental conditions (Penman and Piferrer, 2008; Mank and Avise, 2009; Piferrer et al., 2012). However, in some GSD species, the primary sex can be influenced by environmental factors during sensitive periods of development, during which some individuals can alter their phenotype to the opposite sex without changing their genotype (Ospina-Alvarez and Piferrer, 2008; Shao et al., 2014; Shen and Wang, 2014). In this system, sex is determined by genotype-environment interaction (G × E). In species with this kind of sex-determining system, the genetic mechanism of regular developmental trajectory of gonad is redirected by environmental factors, resulting in a change in sexual phenotype known as environmental sex reversal (Stelkens and Wedekind, 2010; Shao et al., 2014; Holleley et al., 2016). This phenomenon is relatively common in many animal species such as reptiles (Quinn et al., 2007; Holleley et al., 2016), amphibians (Wallace et al., 1999), insects (Vance, 1996; Narita et al., 2007), and fish (Devlin and Nagahama, 2002; Senior et al., 2015), and can rapidly affect the evolution of species (Shao et al., 2014; Holleley et al., 2016). The sex-reversed individuals can affect the number of males and females in a population and may give rise to offspring at skewed sex ratio (Senior et al., 2015). Sex ratio is a key demographic parameter influencing the structure of populations and their reproductive capacity (Penman and Piferrer, 2008). Skewed sex ratios are reflected by sex-biased families (male-biased or female-biased) in a population, resulting in one sex being more abundant than the other sex. Hence, the main concern about skewed sex ratio in species with a G × E sex-determining system is that they may have negative consequences on population dynamics as a result of non-adaptive sex ratio under rapid climate change (Ospina-Alvarez and Piferrer, 2008; Shao et al., 2014). Understanding the mechanism underlying G × E is therefore an important research interest related to the conservation of natural populations and livestock species in order to develop counterstrategies for dealing with unforeseeable ecological consequences.

Zebrafish (*Danio rerio*), a small tropical fish, is an excellent research model animal to study the mechanism of sex determination and the effect of environmental changes on gonadal differentiation in vertebrates (Liew and Orban, 2014; Ribas and Piferrer, 2014). In the process of sex determination in zebrafish, all individuals initially develop an undifferentiated ovary-like gonadal structure called juvenile ovary, regardless of their actual and final phenotypic sex (Uchida et al., 2002; Wang et al., 2007). The bipotent gonad undergo apoptosis during critical embryonic developmental stages or during larval development and shift from ovarian to testicular differentiation in future males. In contrast, ovarian differentiation continues and oocytes grow to maturity in future females (Uchida et al., 2002; Wang et al., 2007; Liu et al., 2015). Cytogenetic studies and breeding experiments on zebrafish indicated that there are no heteromorphic sex chromosomes in zebrafish, suggesting sex is determined by a polygenic sex determination system (PSD), in which the sex-determining genes are distributed across the genome and sex is determined by combining their alleles (Amores and Postlethwait, 1999; Liew et al., 2012; Liew and Orban, 2014). However, a recent restriction site-associated DNA (RAD) mapping study revealed that there is a difference in the genetic makeup of wild zebrafish populations and domesticated strains: the presence of a chromosomal sex determination system in wild populations (ZZ/ZW) and the loss of the region harboring detectable sex-linked loci in domesticated strains during the domestication process (Wilson et al., 2014). Furthermore, the RAD-tag population genomics study in zebrafish also demonstrated that some female genotypes exhibited male phenotypes in natural populations, suggesting that environmental or genetic factors may cause female-to-male sex reversal (masculinization) (Wilson et al., 2014). A further investigation of environmentally induced sex reversal in a domesticated zebrafish strain exposed to high ambient temperature showed that a subset of heat-exposed animals became masculinized and, interestingly, a subset of females with a normal ovarian phenotype exhibited a male-like gonad in response to elevated temperature (Ribas et al., 2017a). In our recent studies of G × E effect on phenotypic plasticity in zebrafish, we found sex ratio variation among different zebrafish families and heat-induced masculinization in response to high temperature (Hosseini et al., 2019a; 2019b), supporting the PSD system and environmentally induced sex reversal in domesticated zebrafish strains in agreement with previous studies (Abozaid et al., 2011, 2012; Liew et al., 2012; Ribas et al., 2017a). A multi-generation selection experiment on sex ratio variation in a large number of zebrafish families revealed very similar offspring sex ratio between repeated crosses from the same breeding pair in sex-biased families, suggesting sex in zebrafish is a heritable trait (Liew et al., 2012). A later investigation of the effect of elevated temperature on zebrafish sex also demonstrated the similarity of progeny sex ratio between repeated crosses of the same breeding pair and showed that the occurrence of changes in sex ratio caused by increased temperature is family specific (Ribas et al., 2017a). Wide variation of sex ratio among different zebrafish families (family-biased sex ratio) and sex reversal in response to environmental conditions during the critical developmental time window in several studies led to the conclusion that sex determination in zebrafish is a PSD system and sex is determined by G × E. However, the genetic mechanisms underlying family-dependent sex ratio in zebrafish and the effect of environmental temperature on their genetic makeup have not yet been fully elucidated.

Sexual development and plasticity of phenotypic sex induced by environmental factors can be influenced by a dynamic epigenetic landscape of chromatin modification during sexual differentiation (Piferrer, 2013). Epigenetic mechanisms can integrate genomic and environmental factors to generate a particular phenotype in response to the internal or external stimulus through changes in the transcriptional activity of genes (Cavalieri and Spinelli, 2017; Piferrer et al., 2019). Methylation of DNA is one of the most important heritable epigenetic modifications in vertebrates, in which a methyl group is added to the 5′ position of cytosine next to a guanine (CpG) by a group of enzymes called DNA methyltransferases (DNMTs) (Bird, 1986; Wu et al., 2011; Piferrer, 2013). Previous studies have reported the importance of epigenetic mechanisms on sexual development, particularly in species with G × E such as in European sea bass (Navarro-Martin et al., 2011), half-smooth tongue sole (Shao et al., 2014), and Nile tilapia (Sun et al., 2016). However, a comprehensive assessment of the function of epigenetic regulation on vertebrate sexual development and its impact on sexual plasticity is far from being clear even in the widely used research model animal, the zebrafish (Shao et al., 2014; Ribas et al., 2017b). In light of global climate change, the effect of epigenetic modifications such as DNA methylation on sexual plasticity in thermosensitive species using the zebrafish as a research model animal is therefore well worth investigating. The mechanisms of epigenetic regulation of sex determination in family-biased sex ratio in zebrafish has not yet been studied. Therefore, in this study, we performed genome-wide DNA methylation in zebrafish gonad to address the effect of epigenetic modifications on sex ratio variation of sex-biased families (male-biased and female-biased) and phenotypic sexual plasticity in response to environmental temperature.

## Materials and Methods

### Animal Husbandry of Parental Generation

The DDR zebrafish strain (Von Hertell et al., 1990; Hosseini et al., 2019a), a laboratory strain, was used in this study. This strain was provided by the Company Aquafarm Ryba Zeven, GmBH (Zeven, Germany) in 1990 (Von Hertell et al., 1990) and were kept in the recirculation systems of aquaculture facilities at the University of Goettingen according to the institutional guidelines on the use of animals for research purpose. The broodstocks were kept at 28 ± 0.5°C and maintained under a 12-h light/12-h dark photoperiod with dissolved oxygen around 7 mg/L and pH value of water 7.4 ± 0.2. The fish were fed twice daily with commercial dry food (Tetramine Junior, Germany) for zebrafish and freshly hatched *Artemia salina* nauplii. Temperature, pH, and dissolved oxygen were monitored daily, whereas other water quality parameters such as ammonia and nitrite were checked periodically to ensure that they were within the appropriate range.

### Experimental Design for Producing Families and Their Rearing Conditions

**FIGURE 1:**
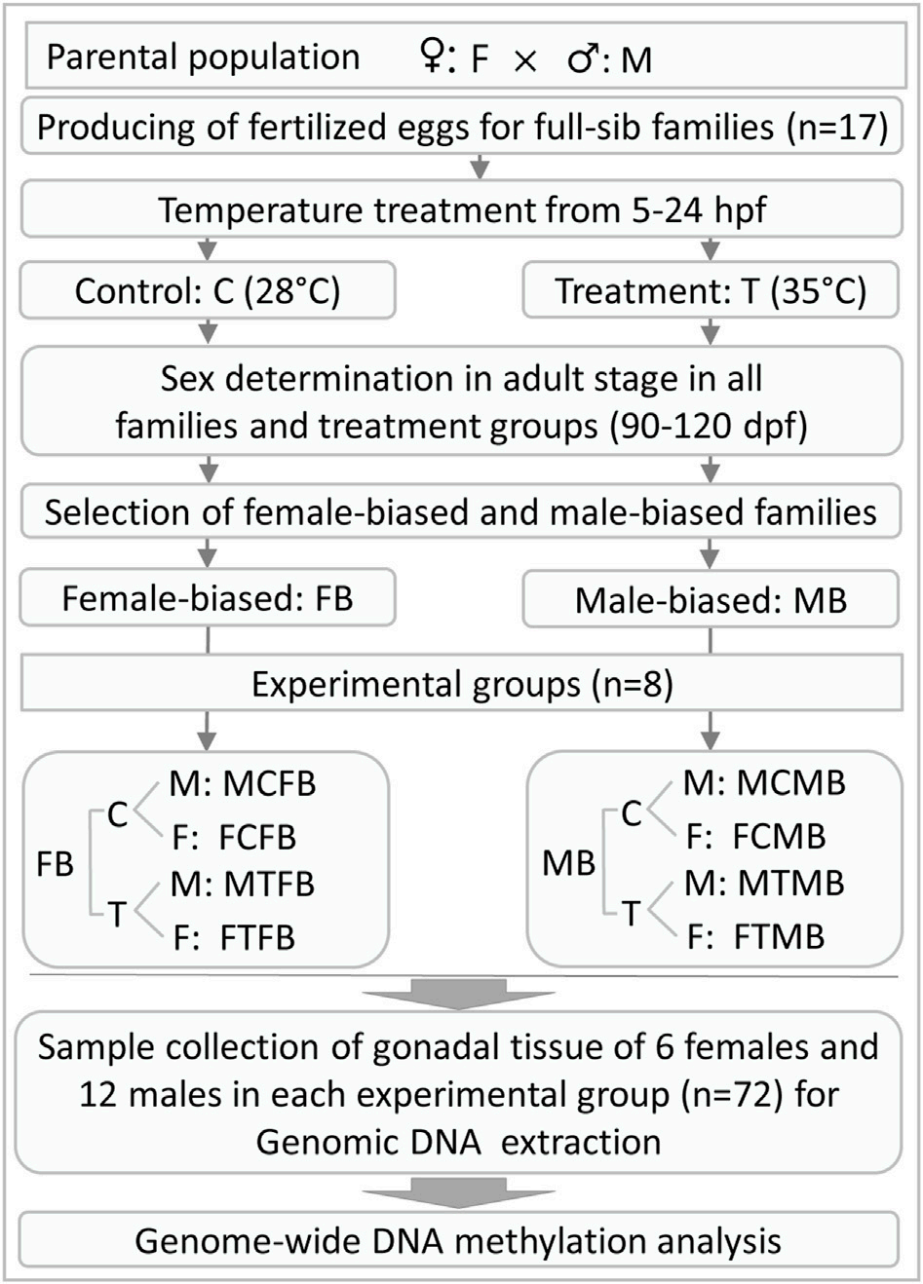
General overview of experimental design and gonadal tissue sample collection for RRBS analysis in this study. hpf, hours post fertilization; dpf, days post fertilization.

In this experiment, the fertilized eggs of 17 full-sib zebrafish families derived from parental generation were submitted in equal proportion to two different temperature incubations: 1) control group was kept at a constant temperature of 28°C, the standard rearing temperature for zebrafish (Detrich et al., 2004; Ribas and Piferrer 2014), throughout the experiment, 2) high incubation temperature group (treatment group) was exposed to the high water temperature of 35°C during embryogenesis from 5 to 24 hours post fertilization (hpf), which is a crucial time window for gonadal development during embryogenesis in zebrafish (Kimmel et al., 1995; Abozaid et al., 2011; Hosseini et al., 2019a). The temperature of the treatment group applied in this study was in accordance with previously reported studies (Abozaid et al., 2011; Hosseini et al., 2019a, 2019b; Valdivieso et al., 2020). The treatment group was returned to the control temperature of 28°C after treatment. The water temperature of the treatment group was changed gradually to avoid heat stress. The larvae of each family were maintained separately in 3-L tanks (Aqua Schwarz GmbH, Germany) under a photoperiod of 12-h light and 12-h darkness and fed three times a day with commercial food for zebrafish (Tetramine baby, Germany) and *Artemia salina* nauplii. Feeding started at 5 days post fertilization (dpf), after the yolk sac was absorbed. At this stage, the larvae of each family were transferred in 7-L tanks. High rearing density during gonadal differentiation in zebrafish (around 15–45 dpf, Ribas et al., 2017c) can influence the sex ratio variation. The appropriate density during this developmental period without significant effects on masculinization was determined to be less than 20 fish per litre (Ribas et al., 2017c). Therefore, we applied the appropriate density within the tanks in this study, as recommended by Ribas et al. (2017c). In the case of a high number of animals within a family, we increased the number of tanks to avoid density-induced masculinization (Supplementary Table 1, S1). The temperature of all experimental groups was measured daily to ensure the accuracy of the experiment. All other husbandry facilities, fish management and water quality control, and animal care for the families produced in this study were identical to our previous study (Hosseini et al., 2019a). After treatment, all experimental groups were then kept under the same conditions until sexual maturity in the adult stage (90–120 dpf). The phenotypic sex of all individuals from both experimental groups was determined at about three and a half months of age to be sure that the phenotypic sex of all animals is distinguishable. In the case of unclear phenotypic sex, microscopic examination of the gonad was used to recognize the sex of individuals. An overview of the experimental design and sample collection in this study is illustrated in Figure 1.

### Statistical Analysis of Sex Ratio in Different Produced Families

Statistical analysis of the sex ratio was performed using the linear logistic model with a binary response variable by applying the GLIMMIX procedure of SAS System 9.3 (Littell et al., 2006). To characterize the male-biased and female-biased families of zebrafish, the significant differences in sex ratio among the different families in the control group were analyzed using the following model 1:

$$\text{log}\left(\frac{\pi_{i}}{1-\pi_{i}}\right)=\;\mu +\alpha_{i}$$

where $\pi_{i}$ is the probability of being male, μ is the general mean effect, $\alpha_{i}$ is the fixed effect of family.

To select the families for reduced representation bisulfite sequencing (RRBS) analysis in this study, two decision criteria were applied: 1) a high difference in the proportion of males between the families under control condition (model 1), and 2) a high difference in the proportion of males between control and treatment groups within the families. The statistical analysis for differences in the proportion of males with regard to the classification of sex-biased families was carried out for the two selected families (male-biased and female-biased) considering the fixed effect of family, treatment, and family-treatment interaction using the following model 2:

$$\text{log}\left(\frac{\pi_{ij}}{1-\pi_{ij}}\right)=\;\mu +\alpha_{i}+\beta_{j}+\alpha_{i}\times \beta_{j}$$

where $\pi_{ij}$ is the probability of being male, μ is the general mean effect, $\alpha_{i}$ is the fixed effect of family, $\beta_{j}$ is the fixed effect of temperature treatment (j = 1: treatment group at 35°C, j = 2: control group at 28°C), and, $\alpha_{i}\times \beta_{j}$ is the fixed effect of interaction between the main factors. Least squares means were estimated on the logit scale and then back transformed using the inverse link function to the original scale. The significant differences between least squares means were tested using a *t*-test procedure by inclusion of the PDIFF option in the Lsmeans statement.

### Tissue Sample Collection for DNA Extraction

We collected the gonadal tissue samples of 6 females and 12 males in each experimental group: control male, control female, treatment male, and treatment female, in two different zebrafish families (male-biased and female-biased) separately. All animals were at the same age (three and a half months), when the samples were taken. Due to the small size of the male gonad tissue, we collected more male gonad samples to pool the DNA samples of two males in each group in the next step of the laboratory analysis. To this end, a total number of 72 gonad tissue samples from male and female animals were collected for RRBS analysis in this study (Figure 1). For this purpose, the gonadal tissue samples were carefully dissected postmortem from animals and flash frozen in liquid nitrogen after dissection and stored at −80°C for further laboratory analysis.

### DNA Isolation and Preparation of RRBS Libraries

Genomic DNA was extracted from gonad samples using the AllPrep DNA/RNA Mini Kit (Qiagen) with proteinase K and RNase A treatment according to the manufacturer’s recommendation. About 20 mg of the ovary tissue from each individual was used for DNA extraction, while the pooled testes of two animals were used to recover enough DNA. The integrity of intact DNA was assessed on 1.2% agarose gel electrophoresis and the quantity was determined using a NanoDrop spectrophotometer. Two micrograms of DNA sample were spiked-in with 1% DNA control [unmethylated cl857 Sam7 Lambda DNA (Promega)] and digested overnight with 100U MspI (New England Biolabs). The DNA fragments were recovered, end-repaired, A-tailed and ligated to TruSeq DNA adapters (cytosines methylated) using the TruSeq Nano DNA library prep kit (Illumina) according to the manufacturer’s protocols. The adapter-ligated DNA libraries were subjected to bisulfite conversion using the EZ DNA Methylation-Gold Kit according to the manufacturer’s protocols (Zymo Research). The libraries were amplified after bisulfite conversion using the PfuTurbo Cx Hotstart DNA Polymerase kit (Stratagene). The quality of the libraries was assessed on 2100 Bioanalyzer using a high-sensitive DNA chip (Agilent Technologies). The normalized RRBS libraries were multiplexed and parallel sequenced for 121 bp single reads on the HiSeq 2500 system (Illumina) at the sequencing facility of Research Institute for Farm Animal Biology (FBN), Dummerstorf, Germany according to the manufacturer’s recommendations using the HiSeq SR Cluster and HiSeq SBS Kit v4 (Illumina).

### Bioinformatics Analysis

The bcl2fastq2 conversion software v2.19 was used to convert base call (BCL) files into fastq files. The overall quality of sequencing reads was inspected pre- and post-processing using FastQC (Babraham Bioinformatics, United Kingdom). The raw fastq files were pre-processed to remove adapter-like sequence and trim 2 bp artificial filled-in of the blunt-end of both 5′and 3′ using TrimGalore (v.0.6.5). Cleaned reads were mapped to the *Danio rerio* reference genome (GRCz11) downloaded from Ensembl (http://ftp.ensembl.org/pub/release-100/fasta/danio_rerio/dna/) and performed methylation calls using Bismark version 0.22.3 (https://www.bioinformatics.babraham.ac.uk/projects/bismark/). Visualization of mapped data was performed using SeqMonk (https://www.bioinformatics.babraham.ac.uk/projects/seqmonk/).

### Differentially Methylation Analysis

In this study, a total of 1,322,526 CpG sites (raw) were mapped at least in one sample. The raw CpGs were filtered to keep only CpG sites that were covered at least 10 reads and present in all samples. Finally, only 210,009 CpG sites passed the filters and used in downstream analyses. The RRBS analysis was performed in the R-Statistics program (R Core Team, 2015) using the “edgeR” package (Robinson et al., 2010; Chen et al., 2017). To assess the effect of DNA methylation in sex-biased zebrafish families, two different analytical strategies were applied in this study: 1) comparing the methylation profiles of male-biased versus female-biased family within each gonad type in control and treatment conditions (see result in Figure 4, comparisons between families) and 2) comparing the methylation profiles of testes versus ovaries within each family type in control and treatment conditions (see result in Figure 6, comparisons within families). This resulted in eight comparisons, for which we performed genome-wide DNA methylation analysis. Differential methylation analysis was conducted in edgeR, considering the counts for methylated and unmethylated reads as separate observations in order to use both reads in statistical model using generalized linear approach underlying a negative binomial distribution and the likelihood ratio test to identify the differentially methylated CpG sites (DMCs) in different experimental groups in form of log_2_ fold change. To fit the total read count at each genomic CpG site, the generalized linear approach modeled the proportion of methylated reads as an over-dispersed binomial distribution. This method allows direct modeling of the inherent variability of the data and thus possibly more realistic analysis of the data. The CpG sites with low coverage (less than 8) were removed from the downstream analysis based on the quality control proposed by edgeR to avoid unreliable results of methylation levels across all samples (Chen et al., 2017). The standard false discovery rate (FDR) approach of Benjamini–Hochberg (Benjamini and Hochberg, 1995) was applied to account for the multiple testing correction to control a statistical significance. The methylation differences between the compared groups were considered to be statistically significant at FDR < 0.05. In this study, we analyzed differential methylation changes of individual CpG sites (DMCs) on a genome-wide scale, which is a single-base resolution and more informative than region analysis approach (differentially methylated regions, DMRs). We have particularly analyzed DMRs focusing on 2 kb upstream and 1 kb downstream of the transcription start site (TSS), which includes the promoter and 5′ upstream regions. A total of 2,977 out of 6,790 promoter regions accommodating 79,340 CpGs passed the filters and were used in downstream analysis. A mean of 12 CpGs and a median of seven CpGs were detected by our RRBS data in the 3 kb promoter regions. For DMRs analysis, we focused on promoter regions due to clear evidence of general relationships with gene expression and regulation. To investigate the sex-determining genes associated with DMCs, we generated a list of candidate genes, most of which have been reported in our previous transcriptomic study (RNA-Seq) in the zebrafish gonad (Hosseini et al., 2019b), in which the experimental design and procedure were identical to this study. However, integration of DNA methylation and expression levels of these candidate genes was not performed in this study due to the use of gonad samples of different animals. We have then expanded this list here to include the genes involved in epigenetic mechanisms. This list was compiled from literature, ZFIN database, and the NCBI gene database for zebrafish. To this end, a total number of 174 genes were used as selected candidate genes in this study (Supplementary Table 2, S2). This list was employed to further investigate the association between the DMCs and the candidate genes in different experimental groups.

### Functional Annotation Analysis

Functional enrichment analysis was performed to gain insight into the biological interpretations of genes associated with differential methylation in comparisons between the two sex-biased families of zebrafish. We used the genes for which the DMCs (Figures 4, 6) were mapped to the genes at the genomic distance from the methylated CpG sites to the TSS (within a window of 10 kb upstream and downstream to the TSS) for pathway and gene ontology (GO) analysis. Likewise, both enrichment analyses were performed for genes that were differentially methylated in the promoter regions (Supplementary Figure 9). For this purpose, two different approaches were applied. In the first analysis, the differentially methylated genes in male-biased versus female-biased family in the control and treatment groups were used for the gene set enrichment analysis, as illustrated in Figure 4, to find the differences between the male-biased and the female-biased families regardless of the effect of sex. In the second analysis, the differentially methylated genes in testes versus ovaries of male-biased and female-biased families were used, as presented in Figure 6. In the latter approach of gene sets enrichment analysis, in order to find the differences between the male-biased and the female-biased families in interaction with sex, we separately merged the results of annotated pathways and GOs of male-biased and female-biased families in the control and in the treatment group, to identify the unique and nonredundant pathways and GOs for each family. For GO enrichment analysis, topGO package was used to test the enrichment of GO terms by applying Fisher’s Exact test (Alexa and Rahnenfuhrer, 2020). To analyze the overrepresentation of pathways in Reactome database, the Wilcoxon test was computed for each gene set. To this end, the GOs and pathways with the *p*-values less than 0.05 were investigated to be enriched in gene set enrichment analysis. All functional enrichment analyses were performed in R-Statistics program (R Core Team, 2015).

## Results

### Sex Ratio Variation in Different Zebrafish Families in Response to Temperature

**FIGURE 2:**
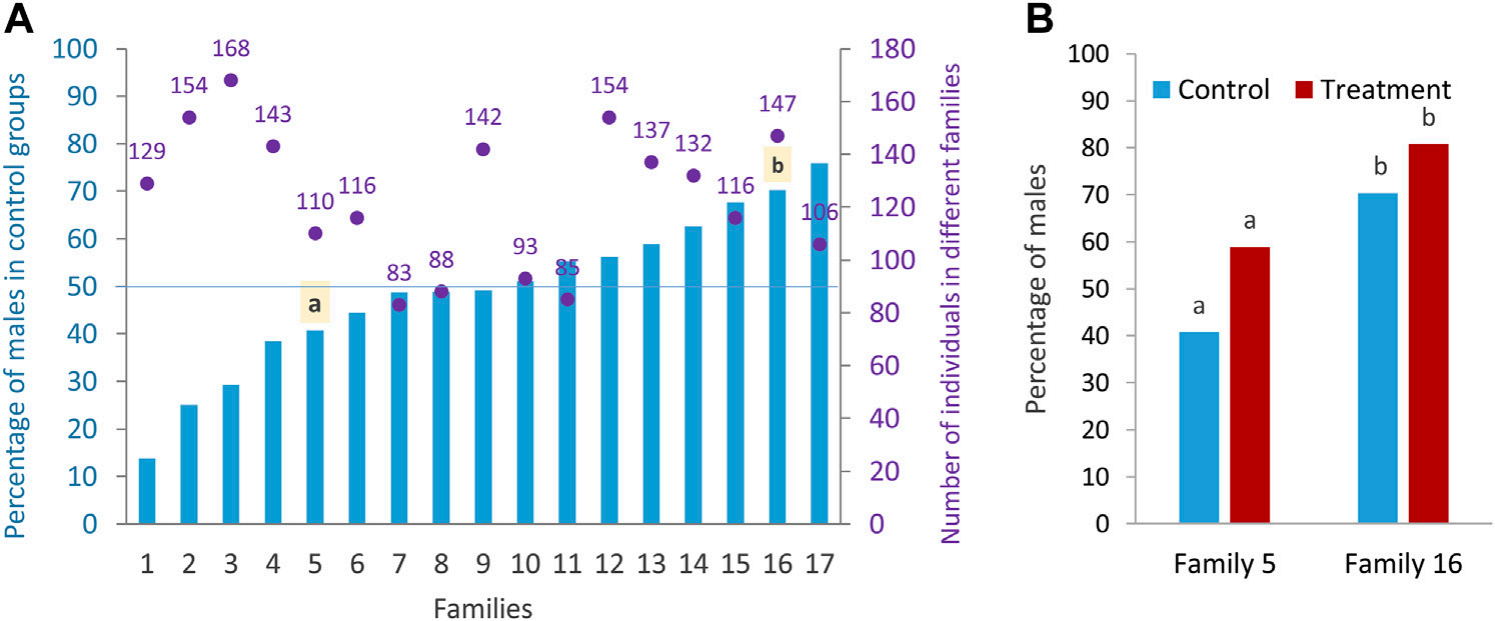
Sex ratio of different zebrafish families. **(A)** Differences in the sex ratio of different families (Lsmeans) in the control group and the total number of sex-determined individuals in the control and treatment of each family. **(B)** Sex ratio of control and treatment groups in the two selected families: family 5 (female-biased family) and family 16 (male-biased family) for RRBS analysis. a–b significant difference between the proportion of male in the control and treatment groups of the two selected families.

In this study, the effect of high incubation temperature (35°C) during embryonic development from 5 to 24 hpf compared to the control temperature (28°C, normal rearing temperature for zebrafish) on sex ratio variation of different zebrafish families (N = 17 families) was examined. In the rest of the paper, the terms “treatment” and “control” are used instead of high incubation temperature and control temperature for simplicity. The results showed a wide range of sex ratio variation between families (Figure 2A), suggesting a PSD in the domestic zebrafish strain used in this study. Based on the sex ratio determined, the families were divided into three categories as described by Liew et al. (2012): 1) female-biased family (less than 40% males in the control group), 2) unbiased family (40%–60% males in the control group), and 3) male-biased family (more than 60% males in the control group). From seventeen different initial families, we selected one female-biased (family 5) and one male-biased (family 16) family to investigate the epigenetic mechanisms of sex determination underlying these two sex-biased families and their response to temperature (see *Materials and Methods*). Considering only the families in the control group (model 1, see *Materials and Methods*) to determine the sex-biased families, and applying a logistic model, there was a significant effect of the factor of family (*p* < 0.0001; *F*-statistic) and a significant difference in the proportion of males between the two selected families (*p* < 0.0015). Considering only the families in the treatment group (model 1), there was also a significant effect of the factor family (*p* < 0.0001; *F*-statistic) and a significant difference in the proportion of males in the treatment group between the two selected families (*p* < 0.006). In the statistical analysis considering only the two selected families (model 2, see *Materials and Methods*), there was a significant difference in the proportion of males independent of temperature effect (49.83 vs. 75.90, *p* < 0.0001). Using the same model for control condition, there was also a significant difference in the proportion of males in the two selected families (40.74 vs. 70.31), which is important in terms of it being a sex-biased family (*p* = 0.0017) (Figure 2B). Considering only the selected families (model 2), a significant difference was found in the proportion of males between the control and treatment groups (*p* = 0.0184) across families. However, the significance level between control and treatment groups was not very pronounced within the two selected families (*p* = 0.0590 and *p* = 0.1454). Since all animals used in this study were kept under the same husbandry conditions and recirculating system with an appropriate stocking density (see *Materials and Methods*), the effect of tank on the level of methylation and consequently on the sex ratio variation within families were not presented.

### Genome-Wide DNA Methylation Landscape and Stratification of Different Experimental Groups

**FIGURE 3:**
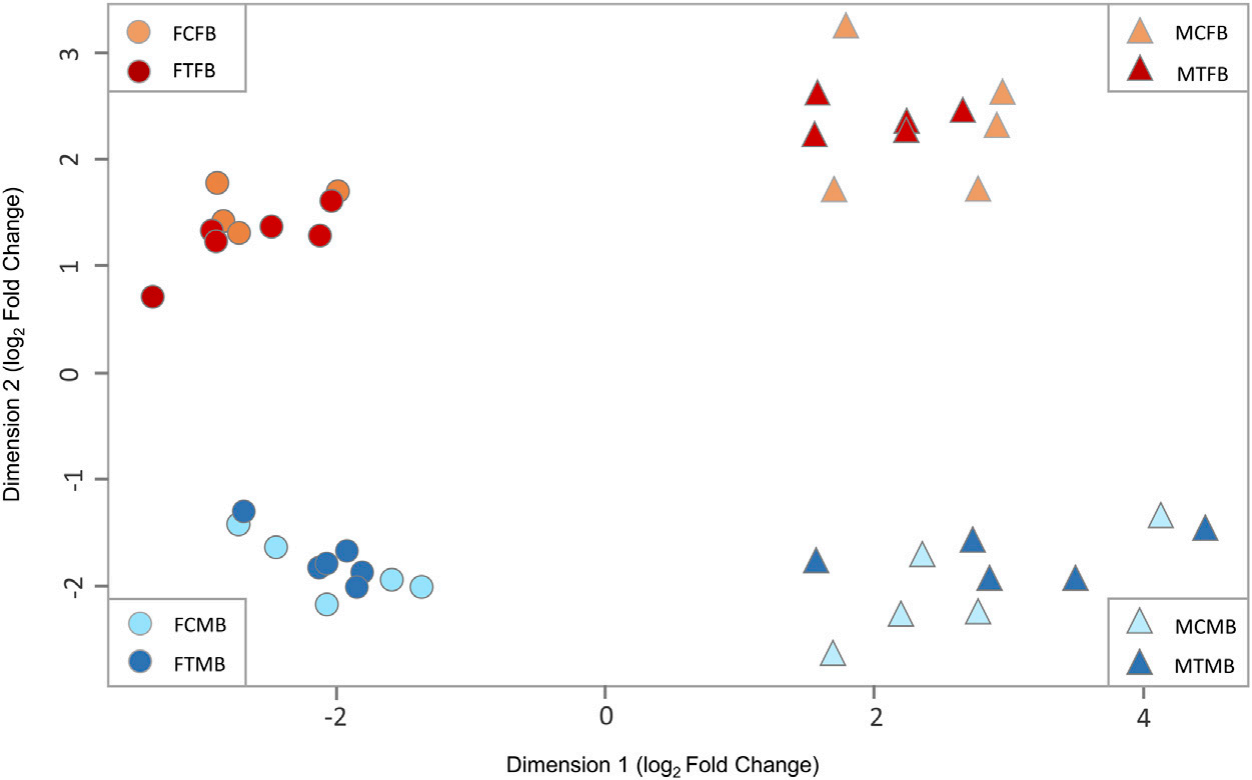
Multidimensional scaling analysis (MDS) of overall DNA methylation profiles in zebrafish gonad. The first dimension (*x*-axis) separates females from males in both sex-biased families. The second dimension (*y*-axis) separates female-biased family from male-biased family. FCFB (female control female-biased), FTFB (female treatment female-biased), MCFB (male control female-biased), MTFB (male treatment female-biased), FCMB (female control male-biased), FTMB (female treatment male-biased), MCMB (male control male-biased), MTMB (male treatment male-biased).

To explore the effect of epigenetic mechanisms on sexual phenotype of sex-biased zebrafish families, we performed multidimensional scaling analysis (MDS) of RRBS datasets to visualize overall differences in DNA methylation (M-value) between individual samples of different experimental groups (Figure 3). The M-value in MDS is calculated from the differences between methylated and unmethylated CpG sites of all individual samples on the log-scale (Chen et al., 2017). The results showed distinct clusters of DNA methylation profiles separating between testes and ovaries in dimension 1 and male-biased and female-biased families in dimension 2 (Figure 3). No clear separation was observed between the control and treatment groups, indicating that there was no pronounced effect of temperature treatment in the overall DNA methylation of CpG sites between individual samples. In addition, we observed no distinct differences between control and treated males in MDS plot. In the study by Ribas et al. (2017a), animals from a female-biased family became 90% male in the treatment group, which was partly due to the effect of high ambient temperature. In the group exposed to the temperature, three or four animals out of ten selected animals for gene expression analysis were expected to be normal males and six or seven animals were expected to be neomales (Ribas et al., 2017a). However, in the same study, a subset of males (2 out of 10, 20%) in the treated group was identified as sex-reversed animals using cluster analysis of the transcriptome profiles. This value corresponds to the 30% of the expected number of neomales in the treatment group. Therefore, 60%–70% of the animals that could be defined as expected neomals had a gonadal expression profile identical to that of the normal males in the control group. In our study, the percentage of males in the treated group was lower than the study of Ribas et al. (2017a) in the two sex-biased families investigated. Hence, the expected number of sex-reversed males in the treated group of our study would be approximately two in the female-biased family and one in the male-biased family out of twelve animals used for RRBS analysis in each family. Therefore, the results of our MDS analysis are consistent with the results of expected masculinization in the treatment group (6 or 7 sex-reversed males out of 10) of the study of Ribas et al. (2017a), which were not detected in the cluster analysis.

We further considered the methylation pattern in the context of annotated genes and CpG islands (Supplementary Figures 1–3). The variations in the DNA methylation landscape of the zebrafish is similar to that of other species (Cavalieri and Spinelli, 2017; Mayne et al., 2020). The methylation level is relatively low around the TSS and increases along up- and down-stream distances away from TSS, as illustrated in Supplementary Figure 1. The pattern is similar between sex-biased families and temperature groups, but differs slightly between testes and ovaries (Supplementary Figures 1A,B). The conservation of the DNA methylation landscape is also found over the gene features and CpG islands. The methylation level increases in the gene body relative to the TSS and decreases at the 3′UTR (Supplementary Figure 2). The methylation level within the CpG island is relatively low compared to 10 kb surrounding regions (Supplementary Figure 3).

### Differentially Methylated CpG Sites in Male-Biased and Female-Biased Families

**FIGURE 4:**
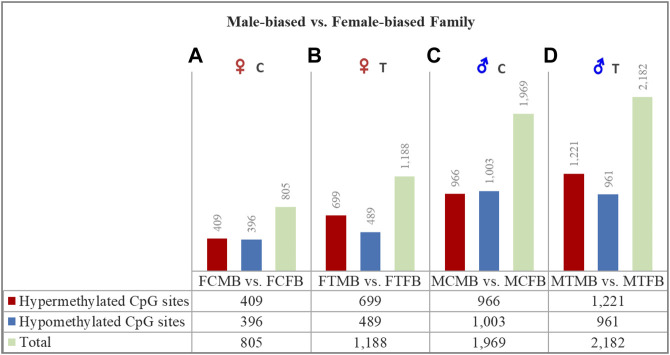
Differentially methylated CpG sites (DMCs) in zebrafish gonad of male-biased versus female-biased family. Bar charts illustrate DMCs in ovaries in control **(A)** and treatment **(B)** groups of male-biased vs. female-biased family (FCMB vs. FCFB and FTMB vs. FTFB, respectively), and in testes in control **(C)** and treatment **(D)** groups of male-biased vs. female-biased family (MCMB vs. MCFB and MTMB vs. MTFB, respectively).

To assess the differences in DNA methylation of sex-biased zebrafish families, given the clear clustering pattern in Figure 3, we compared the methylation profiles of male-biased versus female-biased family within each gonad type in control and treatment conditions in different experimental groups: female control male-biased versus female control female-biased (FCMB vs. FCFB), male control male-biased versus male control female-biased (MCMB vs. MCFB), female treatment male-biased versus female treatment female-biased (FTMB vs. FTFB), male treatment male-biased versus male treatment female-biased (MTMB vs. MTFB) (Figure 4; Supplementary Figure 4; and Supplementary Table 3, S3–6).

The overall results of differentially methylated CpG sites (DMCs) showed that the male-biased family exhibited a higher number of hypermethylated CpGs than the female-biased family in both ovaries and testes in control and treatment groups (FDR < 0.05, Figure 4). Our close observation of differences between the two sex-biased families in each gonad type revealed that a total of 805 CpGs in the control group and 1,188 CpGs in the treatment group were differentially methylated in the ovaries. The same comparison between the two sex-biased families showed that a total of 1,969 CpGs in the control group and 2,182 CpGs in the treatment group were differentially methylated in the testes. Of these DMCs, the number of hypermethylated CpGs in the testes were greater than the ovaries in both control (966 vs. 409) and treatment (1,221 vs. 699) groups. Considering the effect of temperature on DMCs compared between the two sex-biased families revealed more hypermethylated CpGs in the treatment group than the control group in both ovaries (699 vs. 409) and testes (1,221 vs. 966).

### Chromosome-Wise Distribution of Methylated CpG Sites and Their Associated Genes

**FIGURE 5:**
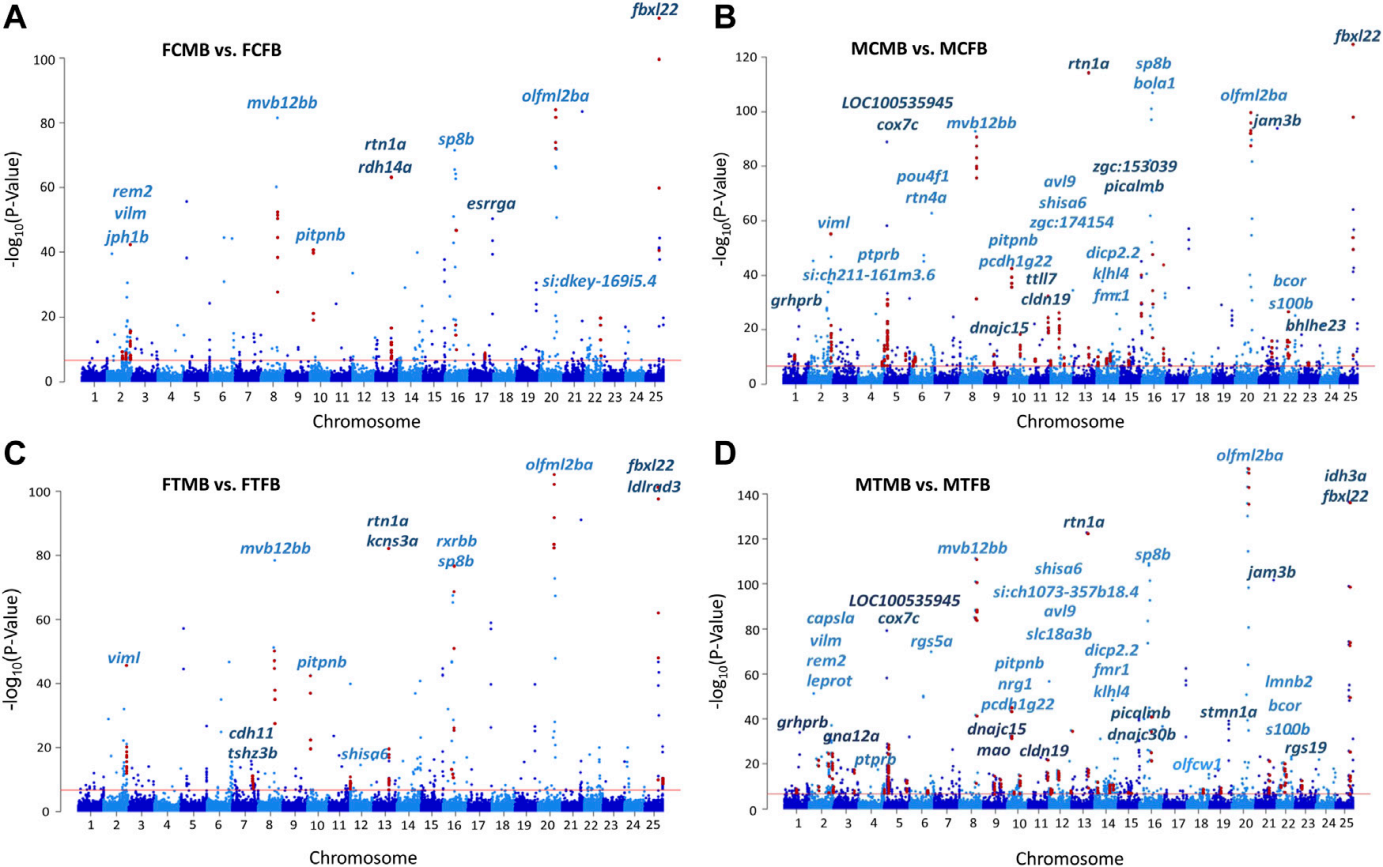
Chromosomal distribution of differentially methylated CpG sites (DMCs) in zebrafish gonad. **(A)** female control male-biased versus female control female-biased (FCMB vs. FCFB); **(B)** male control male-biased versus male control female-biased (MCMB vs. MCFB); **(C)** female treatment male-biased versus female treatment female-biased (FTMB vs. FTFB); **(D)** male treatment male-biased versus male treatment female-biased (MTMB vs. MTFB). Each dot represents a CpG site with genomic position on the *x*-axis and –log_10_ (*p*-value) for differentially methylation between different experimental groups on the *y*-axis. Red dots represent the genomic distribution of DMCs associated with significantly differential genes comprising at least four methylated positions (fifth percentile of distribution). The corresponding gene names are displayed on top of the chromosomes. Red line represents the significance at Bonferroni threshold < 0.05.

The chromosome-wise distribution of DMCs in male-biased versus female-biased family of zebrafish is shown in Manhattan plots (Figure 5). The genes associated with the genomic location of significant DMCs (family-biased genes) with at least four methylated positions are mapped across the chromosomes of the compared experimental groups. This value approximately corresponds to the fifth percentile of the distribution of the top significant methylated positions for all four comparisons. The plots revealed that a few genes (N = 7) were common to all compared groups, whereas the majority of genes (N = 47) associated with the top significant methylated CpG sites differed among these groups. Pairwise comparison between testes and ovaries in male-biased versus female-biased family revealed that the genes associated with the top significant DMCs was greater in the testes than ovaries in both control and treatment groups, as also shown in Figure 4. Consideration of the differentially methylated genes in the control group revealed 29 non-common genes, when comparing FCMB vs. FCFB with MCMB vs. MCFB in the control group (Figures 5A,B). However, in the treatment group, 36 genes differed between FTMB vs. FTFB and MTMB vs. MTFB (Figures 5C,D). Comparison of the distribution of differentially methylated loci mapped to genes in the ovaries of the control and treatment groups (Figures 5A,C) indicated the differences on chromosomes 7, 12, 17, and 22, whereas these differences in the testes were found on chromosomes 3, 18, and 19 (Figures 5B,D). The names of the correspondence genes in each compared group are shown in Figures 5A–D.

We further investigated the overlap between genes associated with DMCs and reproduction-related genes and genes involved in epigenetic mechanisms, which are referred to as candidate genes in this study (*Materials and Methods*). The results showed a subset of them were significantly differentially methylated in different experimental groups in male-biased versus female-biased family of zebrafish (Supplementary Table 4, S7). Among them, two methyltransferase genes (*trmt10c* and *shmt2*) and one chromatin-related gene (*hmgn3*) were significantly differentially methylated in four compared groups. Furthermore, some of the important reproduction-related genes (e.g., *gdf9, star, nr5a2, piwil1, fmr1, sox3, lef1*) were identified in testes and ovaries of male-biased compared to female-biased family in both control and treatment groups. Of these, *fmr1*, which was also observed on the chromosome 14 in the MCMB vs. MCFB and MTMB vs. MTFB in Manhattan plots (Figures 5B,D), showed more DMCs in the testes than ovaries.

### Differentially Methylated CpG Sites in Testes Versus Ovaries Within Sex-Biased Families

**FIGURE 6:**
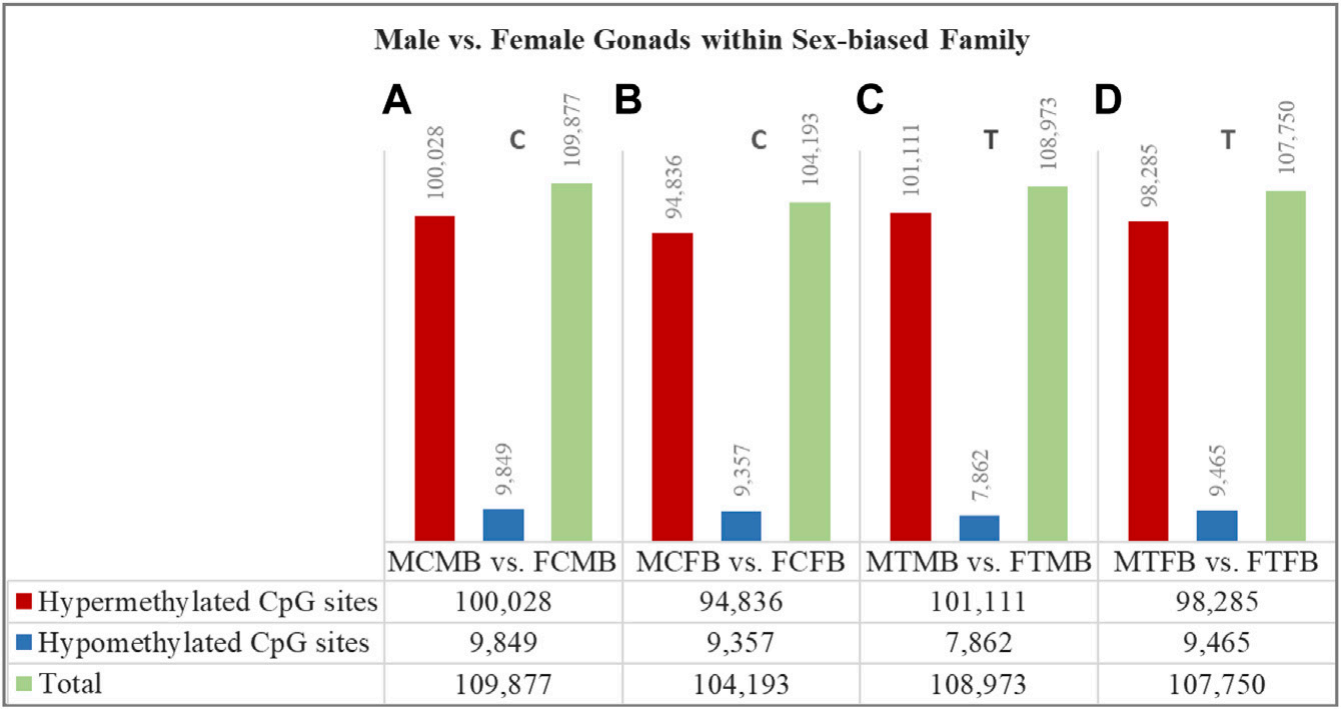
Differentially methylated CpG sites (DMCs) in testes versus ovaries within male-biased and female-biased zebrafish families. Bar charts illustrate DMCs in testes vs. ovaries in control group of male-biased **(A)** and female-biased **(B)** families (MCMB vs. FCMB and MCFB vs. FCFB, respectively), and in testes vs. ovaries in treatment group of male-biased **(C)** and female-biased **(D)** families (MTMB vs. FTMB and MTFB vs. FTFB, respectively).

To understand the epigenetic mechanisms underlying plasticity of sexual development in zebrafish, we compared testes versus ovaries within the two sex-biased families in control and treatment conditions in different experimental groups: male control male-biased versus female control male-biased (MCMB vs. FCMB), male control female-biased versus female control female-biased (MCFB vs. FCFB), male treatment male-biased versus female treatment male-biased (MTMB vs. FTMB), male treatment female-biased versus female treatment female-biased (MTFB vs. FTFB) (Figure 6; Supplementary Figure 5; and Supplementary Tables 5–8, S8–11).

The overall results showed a remarkable difference of DMCs in testes versus ovaries within both sex-biased families, where the DNA methylation landscape of the testes exhibited strikingly higher number of hypermethylated CpGs than the ovaries in both male-biased and female-biased families in control and treatment groups (FDR < 0.05, Figure 6). Further close observations within each family revealed that a total of 109,877 CpGs in the control group and 108,973 CpGs in the treatment group were differentially methylated in the male-biased family. Similarly, a total of 104,193 CpGs in the control group and 107,750 CpGs in the treatment group were differentially methylated in the female-biased family. Among these methylated sites, the number of hypermethylated CpGs in testes versus ovaries of male-biased family was greater than the female-biased family in the control (100,028 vs. 94,836) and treatment (101,111 vs. 98,285) groups. Considering the effect of temperature on DMCs within both sex-biased families demonstrated more hypermethylated CpGs in the treatment group than the control group of both male-biased (101,111 vs. 100,028) and female-biased (98,285 vs. 94,836) families.

### Genes Associated With DNA Methylation Changes in Male-Biased and Female-Biased Families

**FIGURE 7:**
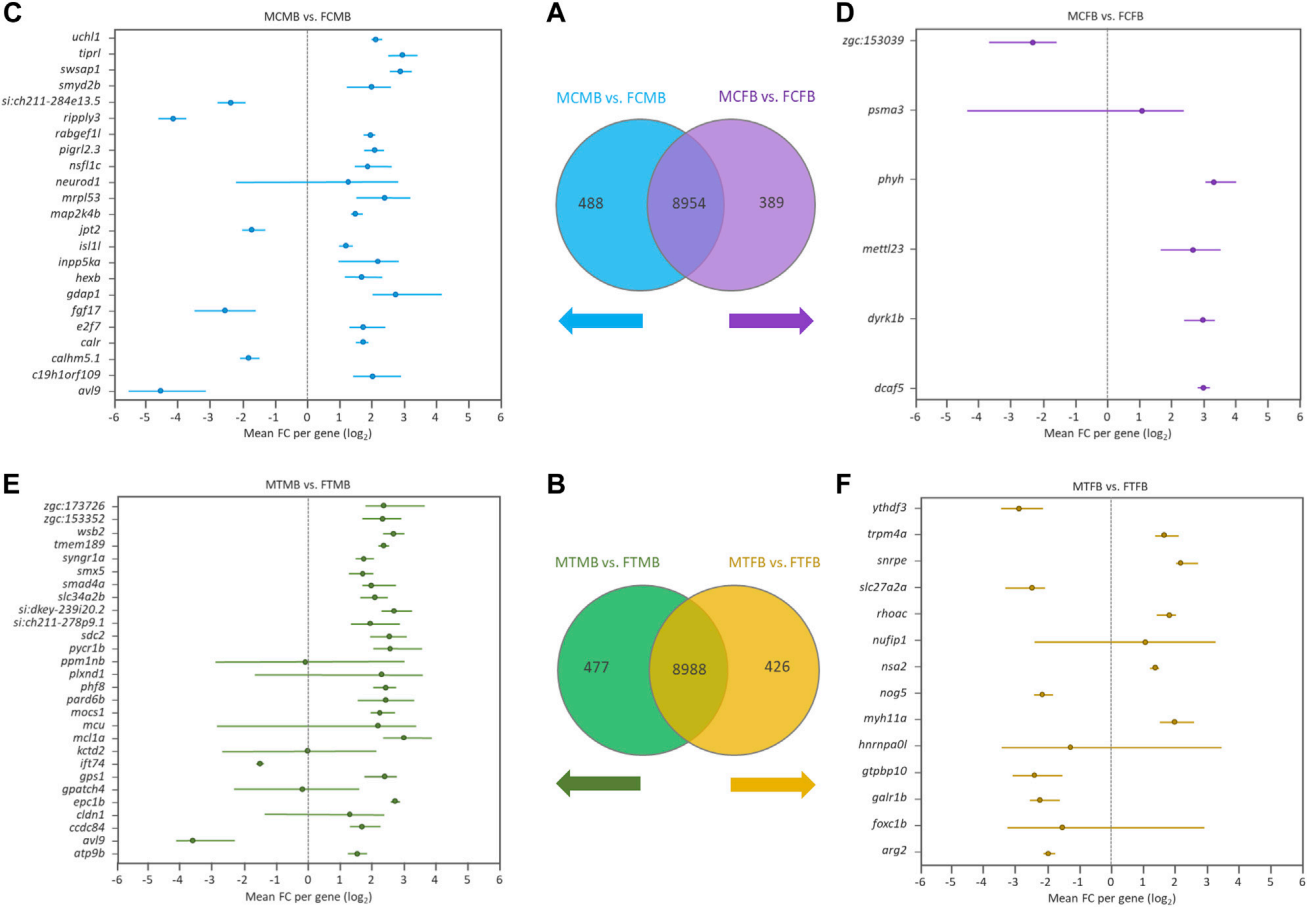
Genes associated with the significantly differentially methylated CpG sites comparing testes versus ovaries within sex-biased families. Venn diagrams illustrate the differences between the male-biased and the female-biased families in the control **(A)** and treatment **(B)** groups. Forest plots represent the unique genes in each sex-biased family, as classified in the Venn diagram, associated with the differentially methylated sites, indicating a range of at least four methylated positions (fifth percentile of distribution) in different experimental groups: **(C)** male control male-biased versus female control male-biased (MCMB vs. FCMB); **(D)** male control female-biased versus female control female-biased (MCFB vs. FCFB); **(E)** male treatment male-biased versus female treatment male-biased (MTMB vs. FTMB); **(F)** male treatment female-biased versus female treatment female-biased (MTFB vs. FTFB). The *x*-axis represents the differences mean fold change (log_2_ FC) per gene.

To find the effect of DNA methylation changes on genes in male-biased and female-biased families, we annotated the DMCs with nearest genes comparing testes versus ovaries, resulting in the detection of unique genes (family-specific genes) in each family and common genes in overlap between the two sex-biased families (Figures 7A,B). The results of Venn diagram showed 488 unique genes in the male-biased family and 389 unique genes in the female-biased family in the control group (Figure 7A). The same comparison in the treatment group revealed 477 and 426 unique genes in the male-biased and female-biased family, respectively (Figure 7B). The unique genes of each sex-biased family with a range of at least four DMCs are shown in the Forest plots (Figures 7C–F). This value corresponds approximately to the fifth percentile of the distribution of the number of the significant methylated positions for all compared groups. The plots showed the range of variation (minimum and maximum values) and the mean values in the level of methylation (log_2_ FC) for different positions associated with unique genes in each sex-biased family. The result indicated that the number of significant methylated unique genes was higher in male-biased (23 genes) than female-biased (6 genes) family in the control group. The same trend was observed in the treatment group, 28 genes in the male-biased and 14 genes in the female-biased family.

Investigation of the overlap between candidate genes and the methylated unique genes in each sex-biased family (Figures 7A,B) revealed the differential methylation of a few genes in all compared groups (data not shown). The number of DMCs associated with these candidate genes was low, so that the validation of their association is not robust enough.

**FIGURE 8:**
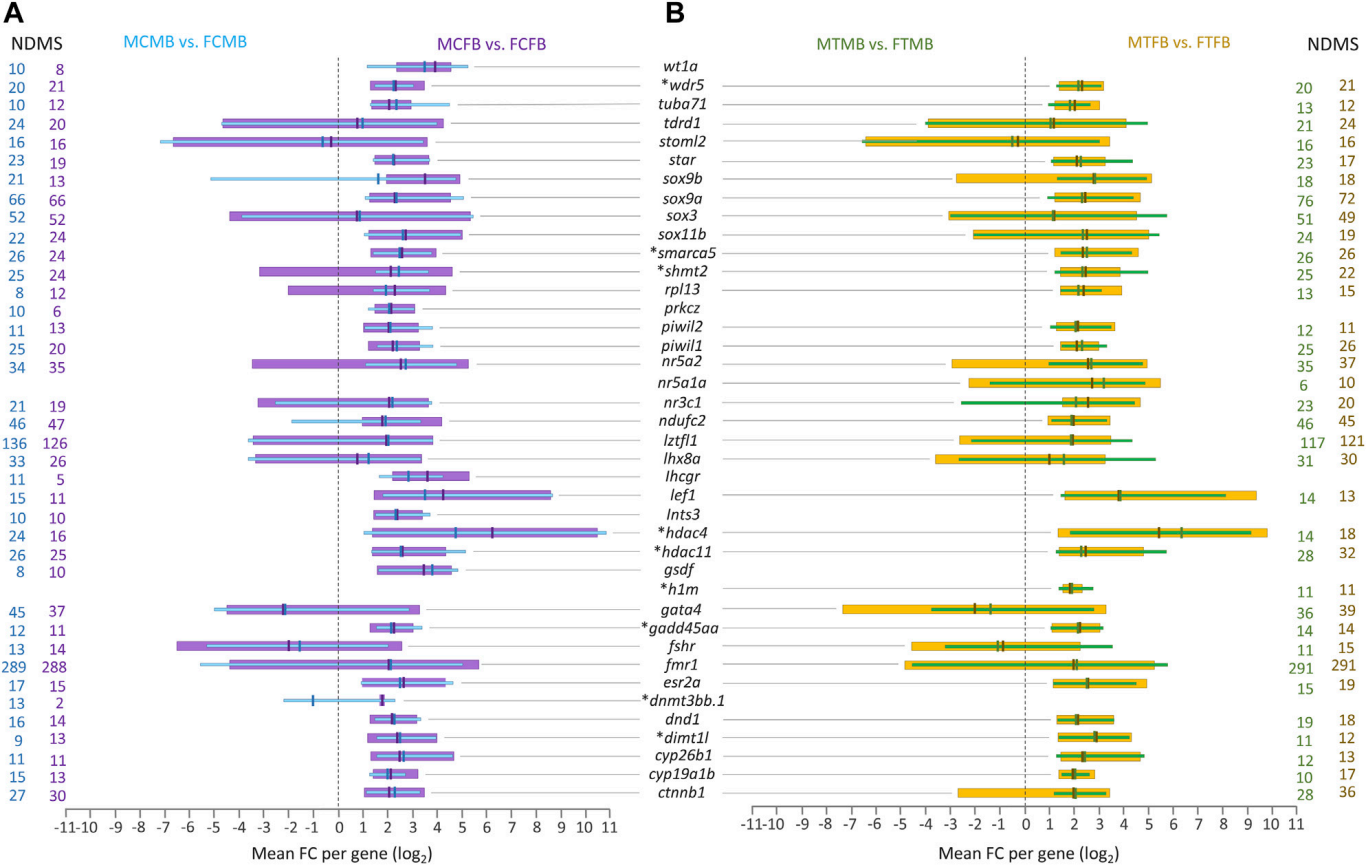
Candidate genes associated with significantly differentially methylated CpG sites in overlap between male-biased and female-biased families. **(A)** male control male-biased versus female control male-biased (MCMB vs. FCMB) in overlap with male control female-biased versus female control female-biased (MCFB vs. FCFB); **(B)** male treatment male-biased versus female treatment male-biased (MTMB vs. FTMB) in overlap with male treatment female-biased versus female treatment female-biased (MTFB vs. FTFB). This figure illustrates a range of at least 10 methylated positions per gene, where the number of differentially methylated sites (NDMS) for each gene is shown with the corresponding group colour. Genes involved in epigenetic mechanisms are marked with an asterisk (*). Other genes are reproduction-related genes. The *x*-axis represents the differences in mean fold change (log_2_ FC) per gene.

Further investigation of the overlap between candidate genes and common genes in the two sex-biased families, 8,954 genes in the control group (Figure 7A) and 8,988 genes in the treatment group (Figure 7B), demonstrated that most of them were significantly differentially methylated in both sex-biased families (Figures 8A,B).

The level of significantly differentially methylated candidate genes (log_2_ FC) illustrated the range of minimum to maximum value variation and the mean values in the compared groups corresponding to at least ten DMCs for each gene. Our findings showed that many of the reproduction-related genes (e.g., *sox9a, sox3, nr5a2, ndufc2, lztfl1, lhx8a, gata4, fmr1, ctnnb1*) were highly methylated in the gonads of male-biased and female-biased families in both control and treatment conditions. Of these methylated genes, *fmr1* and *lztfl1* were associated with a strikingly high number of DMCs in all compared groups, over 288 genomic positions with *fmr1* and 117 with *lztfl1*. Moreover, the methylation of some other reproduction-related genes, *wt1a, prkcz, lhcgr, ints3, gsdf*, was identified in the control group of both sex-biased families (Figure 8A), in which they were not differentially methylated in the treatment group. However, differential methylation of only one sex gene, *nr5a1a*, was found in the treatment group of both families (Figure 8B), whose methylation was not detected in the control group.

Extending the consideration of candidate genes involved in epigenetic mechanisms in overlap between the two sex-biased families revealed that a subset of them, including *wdr5, smarca5, shmt2, hdac4, hdac11, h1m, gadd45aa, dnmt3bb.1,* and *dimt1l*, were differentially methylated in different compared groups, where *dnmt3bb.1* and *h1m* were identified only in the control and in the treatment group, respectively (Figures 8A,B). In general, the corresponding evidence for Figures 7, 8 may not be considered informative and conclusive regarding differences in the methylation levels for a few genes, whose log_2_ FC levels are in both negative and positive directions.

### Differentially Methylated Promoters in Sex-Biased Zebrafish Families

Since the DNA methylation of the promoter region plays an important regulatory function and is often inversely associated with the transcription of the genes, it is an important biological concern to investigate the DNA methylation within gene promoters (Chen et al., 2017). In this regard, we applied a region-wise analysis approach, considering DMRs focusing on 2 kb upstream and 1 kb downstream of the TSS, which includes the promoter and 5′ upstream regions. A total of 2,977 gene promoters were covered in all samples and tested for differentially methylated promoters (DMPs, FDR < 0.05). We first compared the promoters in male-biased versus female-biased family within each gonad type and temperature conditions (Supplementary Figures 6, 7 and Supplementary Table 9, S12–15). The overall results showed that the male-biased family exhibited a higher number of hypermethylated promoters than the female-biased family in both ovaries and testes in control and treatment groups (Supplementary Figure 7). Further observations of the differences between the two sex-biased families in the promoters indicated that the total number of DMPs was higher in the testes than the ovaries in the control (44 vs. 16; Supplementary Figure 7) and treatment (47 vs. 40; Supplementary Figure 7) groups. Consideration of the temperature effect on DMPs comparing male-biased and female-biased families revealed that the DNA methylation changes of the promoters in the treatment groups were greater than those in the control groups.

We then compared the promoters in testes versus ovaries within each sex-biased family in control and in temperature conditions (Supplementary Figures 8, 9 and Supplementary Tables 9, S16–19). The overall results showed a distinct difference of DMPs in testes versus ovaries within both sex-biased families, where the testes exhibited more hypermethylated promoters than the ovaries in both male-biased and female-biased families in control and treatment groups (Supplementary Figure 9). A close observation of DMPs within the male-biased family revealed a total number of 481 hypermethylated promoters in the control group and 492 hypermethylated promoters in the treatment group. Similarly, a total number of 495 hypermethylated promoters in the control group and 478 hypermethylated promoters in the treatment group of the female-biased family was observed. Consideration of the effect of temperature on DNA methylation changes of the promoters in testes versus ovaries revealed a lower total number of DMPs in the treatment group compared to the control group in the male-biased (809 vs. 967) and in the female-biased (913 vs. 958) families, resulting from higher hypomethylated promoters in the treatment group, although the number of hypermethylated promoters was higher than that of hypomethylated promoters in both families.

### Genes Associated With DNA Methylation Changes of Promoters

**FIGURE 9:**
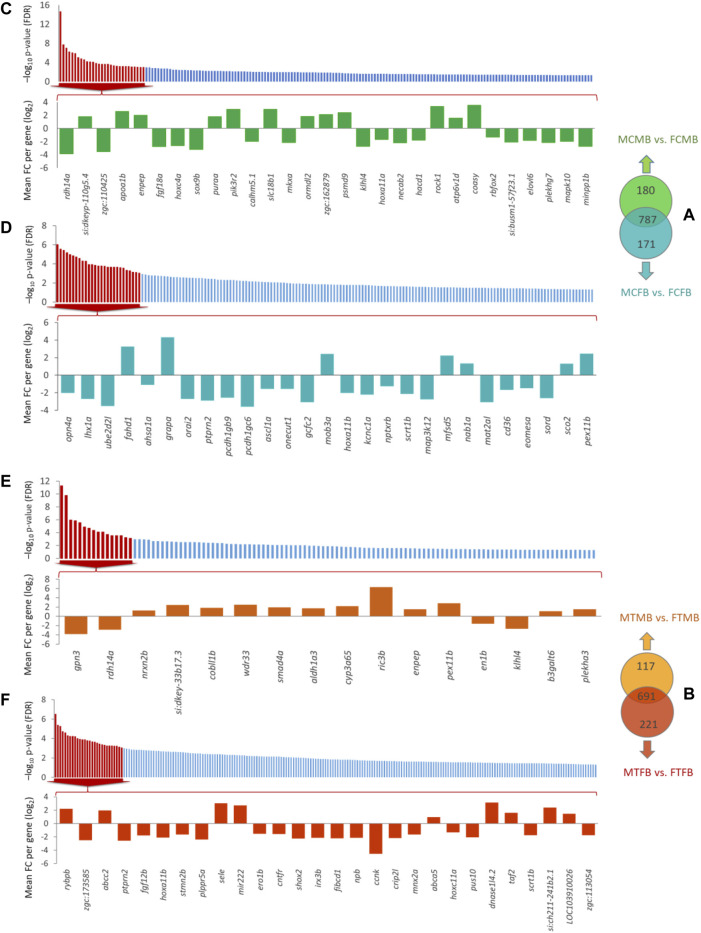
Genes associated with the significantly differentially methylated promoters comparing testes versus ovaries within sex-biased families. Venn diagrams illustrate the differences in methylated gene promoters comparing male-biased and female-biased families in the control **(A)** and treatment **(B)** groups. Bar charts illustrate the level of the significance (FDR < 0.05) for all unique genes in each sex-biased family, and the differentially methylated of top significant unique gene promoters with FDR < 0.001 in male-biased and female-biased families, as classified in Venn diagram, in different experimental groups: **(C)** Male control male-biased versus female control male-biased (MCMB vs. FCMB); **(D)** Male control female-biased versus female control female-biased (MCFB vs. FCFB); **(E)** Male treatment male-biased versus female treatment male-biased (MTMB vs. FTMB); **(F)** Male treatment female-biased versus female treatment female-biased (MTFB vs. FTFB).

To find the effect of DNA methylation changes on gene promoters, we compared testes versus ovaries in male-biased and female-biased families, resulting in the detection of unique genes (family-specific genes) in each family and common genes in overlap between the two sex-biased families (Figures 9A,B). The results of Venn diagram showed 180 unique genes in the male-biased family and 171 unique genes in the female-biased family in the control group, indicating more methylated unique genes in the male-biased family than the female-biased family in the control group (Figure 9A). However, the same comparison in the treatment group revealed considerably more methylated unique genes in the female-biased family (221 genes) than the male-biased family (117 genes) (Figure 9B). The corresponding significance level (FDR < 0.05) of all identified unique genes in each sex-biased family is shown in Figures 9C–F. Of these genes, the top significant unique genes (FDR < 0.001) of the male-biased and the female-biased families in the control and in the treatment groups are shown in the bar charts. The bar charts revealed that a larger number of the top significant unique genes in the control group of the two sex-biased families were hypomethylated (Figures 9C,D). However, in the treatment group, more of the top significant unique genes in the male-biased family were hypermethylated than to the female-biased family (Figures 9E,F).

**FIGURE 10:**
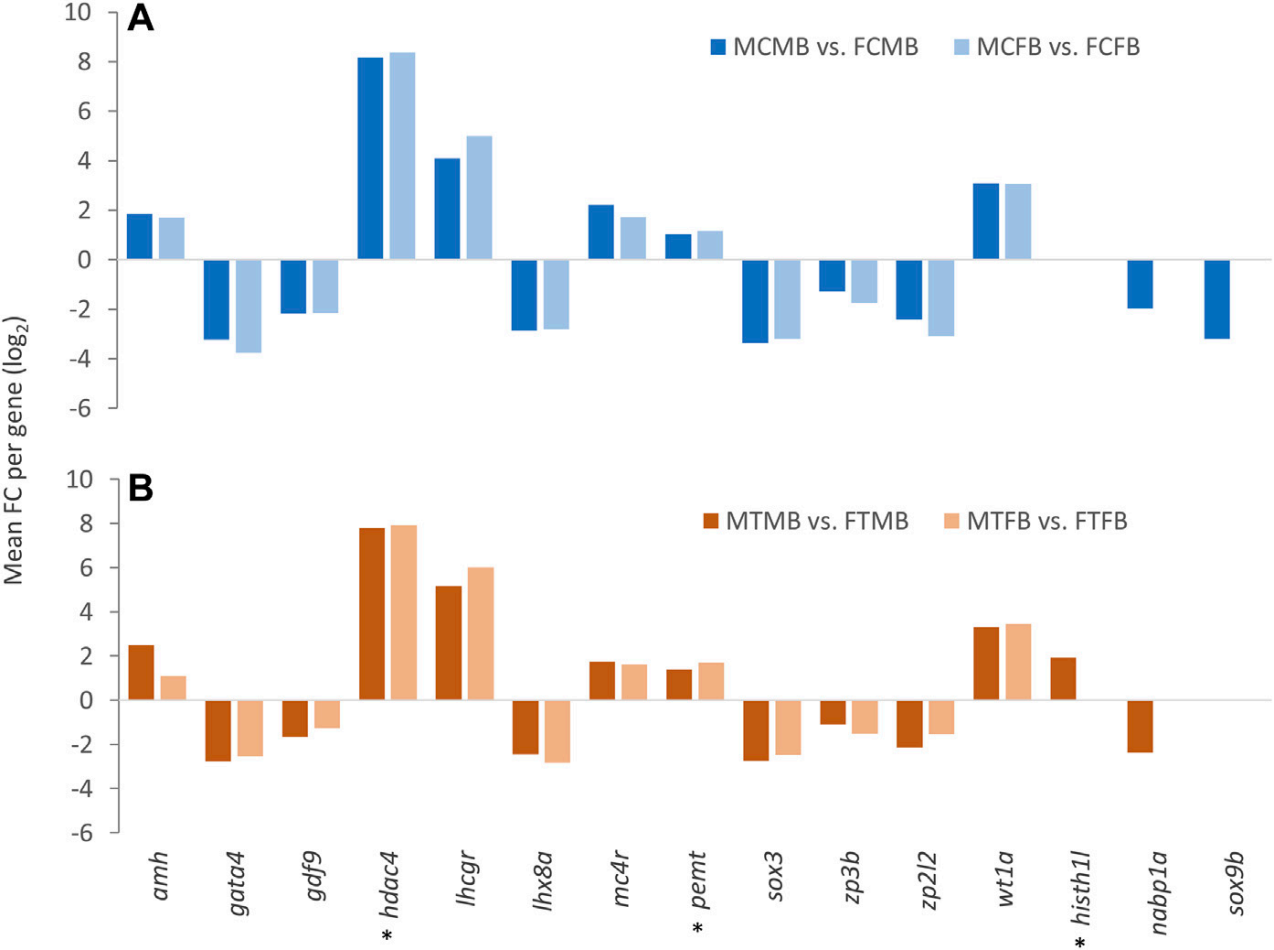
Candidate genes associated with significantly differentially methylated promoters in male-biased and female-biased families. **(A)** male control male-biased versus female control male-biased (MCMB vs. FCMB); male control female-biased versus female control female-biased (MCFB vs. FCFB). **(B)** male treatment male-biased versus female treatment male-biased (MTMB vs. FTMB); male treatment female-biased versus female treatment female-biased (MTFB vs. FTFB). The *x*-axis represents the candidate genes and the *y*-axis indicates mean fold change (log_2_ FC) per gene. Genes involved in epigenetic mechanisms are marked with an asterisk (*). Other genes are reproduction-related genes.

Consideration of the overlap between candidate genes and methylated unique gene promoters in each sex-biased family (Figures 9A,B) revealed the methylation of *nabp1a* and *sox9b* in the control group and *histh1l* and *nabp1a* in the treatment group of only male-biased family, where their methylated promoters were not detected in the female-biased family (Figures 10A,B). A further investigation of the overlap between candidate genes and common genes in both sex-biased families, 787 genes in the control group (Figure 9A) and 691 genes in the treatment group (Figure 9B), showed a subset of candidate genes, including *amh, hdac4, lhcgr, mc4r, pemt, wt1a* exhibited hypermethylated promoters in the two sex-biased families (Figure 10)*.*

### Functional Annotation Analysis of Differentially Methylated Genes

**FIGURE 11:**
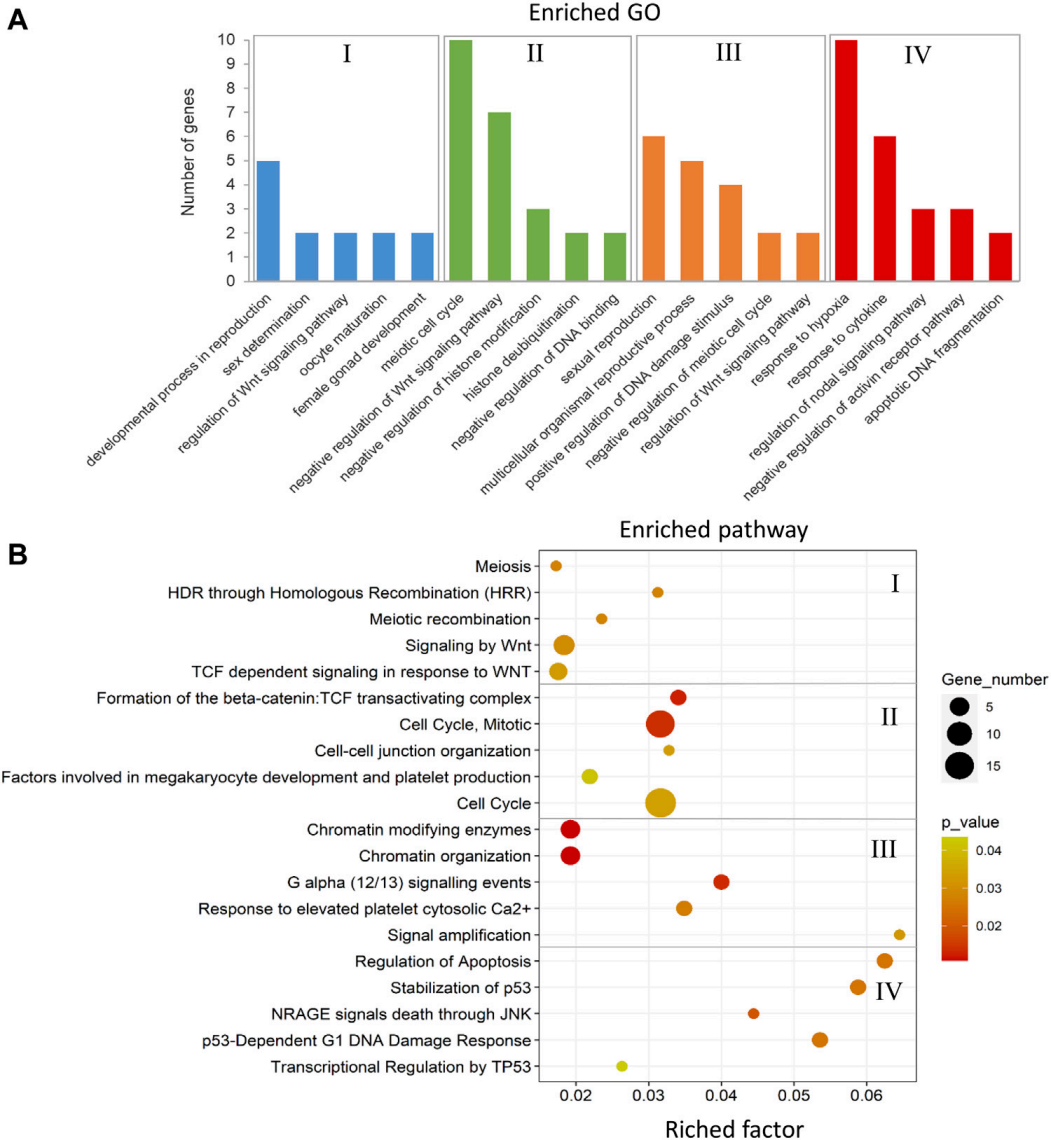
Enriched GOs and pathways in male-biased versus female-biased family. The results of biological functional annotation analysis of genes associated with DMCs in male-biased versus female-biased family in different experimental groups: (I) female control male-biased versus female control female-biased (FCMB vs. FCFB); (II) male control male-biased versus male control female-biased (MCMB vs. MCFB); (III) female treatment male-biased versus female treatment female-biased (FTMB vs. FTFB); (IV) male treatment male-biased versus male treatment female-biased (MTMB vs. MTFB). **(A)** Bar charts represent the significant enriched GOs (*p* < 0.05). The vertical axis represents the most important enriched GOs and the horizontal axis represents the number of significantly differentially methylated genes in each GO term. **(B)** Scatter plots illustrate the significant enriched pathways (*p* < 0.05). The vertical axis represents the enriched pathway categories and the horizontal axis represents the rich factor of the enriched pathways. Rich factor is the ratio of differentially methylated gene number enriched in the pathway to the total gene number in a certain pathway. The size and colour of dots represent the gene number and the range of *p*-values, respectively.

**FIGURE 12:**
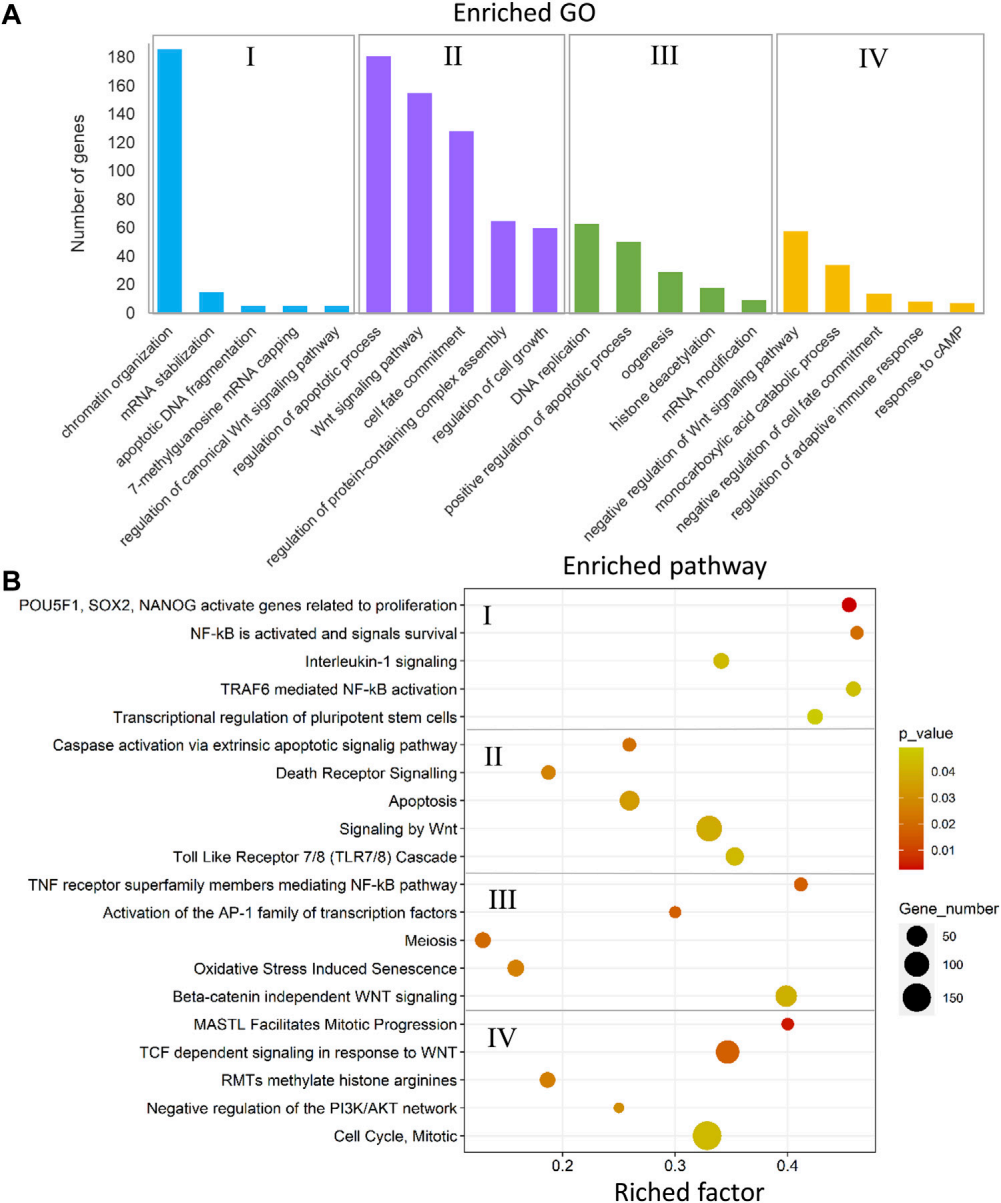
Enriched GOs and pathways in testes versus ovaries. The results of biological functional annotation analysis of genes associated with DMCs in testes versus ovaries within male-biased and female-biased families in different experimental groups: (I) male control male-biased versus female control male-biased (MCMB vs. FCMB); (II) male control female-biased versus female control female-biased (MCFB vs. FCFB); (III) male treatment male-biased versus female treatment male-biased (MTMB vs. FTMB); (IV) male treatment female-biased versus female treatment female-biased (MTFB vs. FTFB). **(A)** Bar charts represent the significant enriched GOs (*p* < 0.05). The vertical axis represents the most important enriched GOs and the horizontal axis represents the number of significantly differentially methylated genes in each GO term. **(B)** Scatter plots illustrate the significant enriched pathways (*p* < 0.05). The vertical axis represents the enriched pathway categories and the horizontal axis represents the rich factor of the enriched pathways. Rich factor is the ratio of differentially methylated gene number enriched in the pathway to the total gene number in a certain pathway. The size and colour of dots represent the gene number and the range of *p*-values, respectively.

The annotated genes associated with DMCs in the comparison between the male-biased and female-biased families of zebrafish (Figures 4, 6) were subjected to functional enrichment analysis (see *Materials and Methods*). The results of the functional analysis represent the biological function of the identified differentially methylated genes in the pathway and Gene Ontology (GO) categories. The important five GOs and pathways (*p* < 0.05) associated with the reproduction and epigenetic mechanisms in zebrafish gonad in the male-biased and female-biased families, based on the presented results in Figures 4, 6, are shown in Figures 11, 12. The complete lists of enriched GOs and pathways are provided in Supplementary Tables 10 and 11.

The enriched GOs and pathways in the male-biased versus female-biased family differed markedly for both testes and ovaries as well as for control and treatment groups (Figure 11 and Supplementary Table 10, S20–27). Consideration of the enriched GOs for testes and ovaries comparing the male-biased versus female-biased family of zebrafish revealed a number of significant GOs such as developmental process in reproduction, sex determination, oocyte maturation, sexual reproduction, and regulation of Wnt signaling pathway for ovaries, and negative regulation of Wnt signaling pathway, negative regulation of histone modification, and apoptotic DNA fragmentation for testes (Figure 11A). The result of the pathway analysis showed the overrepresentation of the important pathways for ovarian development, namely signaling by Wnt, TCF dependent signaling in response to Wnt, and G alpha signaling events. The same analysis revealed the enrichment of several significant pathways for testes development, including cell cycle, regulation of apoptosis, and transcriptional regulation by TP53 (Figure 11B).

The enriched GOs and pathways in the testes versus ovaries revealed a considerable number of different GOs and pathways for each sex-biased family in the control and temperature groups (Figure 12 and Supplementary Table 11, S28–35). The results of unique and nonredundant GOs showed the enrichment of significant GOs for each sex-biased family such as chromatin organization, regulation of canonical Wnt signaling, oogenesis, and histone deacetylation for male-biased family, and Wnt signaling pathway, regulation of apoptotic process, and negative regulation of Wnt signaling pathway for female-biased family (Figure 12A). Similarly, the result of unique and nonredundant pathways revealed the overrepresentation of important pathways involved in zebrafish sex determination in the male-biased family such as NF-κB activated and signals survival, activation of the AP-1 family of transcription factor, and beta-catenin independent Wnt signaling. The overrepresented pathways in the female-biased family indicated enrichment of genes involved in several sex-determining pathways, namely apoptosis, signaling by Wnt, and negative regulation of the P13K/AKT network (Figure 12B).

The results of functional analysis of genes with differentially methylated promoters (Supplementary Figure 8) in testes versus ovaries of the two sex-biased families have been illustrated in Supplementary Tables 12 and 13. Our results revealed the enrichment of GOs involved in reproduction and epigenetic regulatory mechanisms in the male-biased family, namely negative regulation of canonical Wnt signaling pathway, regulation of oocyte development, and negative regulation of chromatin silencing. Likewise, we identified several important reproductive-related GOs in the female-biased family such as reproduction, spermidine acetylation, and regulation of non-canonical Wnt signaling pathway. The result of pathway analysis showed overrepresentation of several reproductive regulatory pathways in the male-biased family such as apoptosis, stabilization of p53, and TNFR2 non-canonical NF-κB pathway. However, in the female-biased family, we found only two pathways, including signaling by Wnt and processing of capped intron-containing pre-mRNA.

## Discussion

### Wide Variation of Sex Ratio Among Zebrafish Families

In this study, we used zebrafish as a model animal to characterize sex ratio variation among different families and compared genome-wide DNA methylation in male-biased and female-biased families to gain new insights into the epigenetic regulatory mechanisms underlying sexual plasticity. Our findings on sex ratio variation among different families of this domesticated zebrafish strain support previous observations of the PSD system in domesticated zebrafish strains (Abozaid et al., 2011, 2012; Ribas et al., 2017a; Hosseini et al., 2019a; Valdivieso et al., 2020). In our previous study with the same temperature treatment conception during embryonic development (Hosseini et al., 2019a), a reduction in survival ability was observed up to 24 hpf compared to the pre-treatment stage at 5 hpf, with a significant lower survival ability in treated group (73.30% ± 0.58% in control vs. 70.19% ± 0.57% in treated group). However, this difference between control and treated groups was not very pronounced (3.1%), indicating that the observed mortality was not due to high temperature during the treatment period (Hosseini et al., 2019a). The same observation was reported in the study by Ribas et al. (2017a), in which different zebrafish families were exposed to high temperature during larval stage. Therefore, the sex ratio observed in different families in this study could not be due to sex-specific mortality.

A breeding experiment in a large number of domesticated zebrafish families revealed that broods derived from the same breeding pair exhibited similar sex ratio between repeated crosses, suggesting that sex in zebrafish is a heritable trait and parental genotypes have a strong influence on offspring sex ratio (Liew et al., 2012). This evidence indicated a genetic sex determination system in domesticated zebrafish strains, which can be influenced by environmental factors. In species with a chromosomal sex determination system, the chance of such sex-biased families occurring is likely to be very low because the sex ratio tends to stay at 1:1 under natural selection (Fisher, 1930), thereby a skewed sex ratio always returns to an equal ratio of males and females in the next generation (Liew et al., 2012; Fryxell et al., 2015). However, a PSD system can interact with environmental factors, resulting in alteration of the individual sexual phenotype and consequently producing offspring with a skewed sex ratio in a population. The PSD system, involving multiple autosomal genes, requires a complex regulatory network for proper sexual development (Liew and Orban, 2014). In these processes, epigenetic regulatory mechanisms including DNA methylation, histone modification, and non-coding RNAs likely contribute to sex determination and reproductive organ development (Piferrer, 2013; Liew and Orban, 2014). Given that a wide variety of sex ratio was observed in different zebrafish families in this study, we propose that an epigenetic mechanism may influence the directions of gonadal development, resulting in skewed sex ratio in the offspring and generating sex-biased families.

### Role of Epigenetic Modification in Regulating Differences Between Sex-Biased Families

The expression of different phenotypes is encoded not only by the genetic information of the DNA sequence, but also by epigenetic modifications of chromatin structures including DNA methylation and alterations of histone proteins that bind DNA to regulate gene expression (Barros and Offenbacher, 2009). The associations between DNA methylation changes and genetic sequence on gene expression between individuals revealed the interdependence of genetic and epigenetic variations on the level of gene expression. The role of DNA methylation can be dependent on genomic and molecular structure of DNA (Gutierrez-Arcelus et al., 2013). In addition, DNA methylation and gene expression levels can be influenced by transcription factors, whose binding levels can be determined by differences in abundance and genetic variants at their binding sites (Gutierrez-Arcelus et al., 2013). Genetic information and epigenetic marks of the genome are not completely separate streams that influence phenotypic expression, but functionally interdependent (Richards, 2006; Richards et al., 2017; Adrian-Kalchhauser et al., 2020). Allelic variants of genes can influence induction of epigenetic marks, whereby the genetically distinct lines of the same species exhibit different effects of parental conditions on the progeny phenotypes (Adrian-Kalchhauser et al., 2020). Inherited gene regulation, which consists of all inherited factors that alter gene expression, from parents to offspring is not fully independent of DNA sequence (Adrian-Kalchhauser et al., 2020). Studies revealed that parental DNA methylation erased during germ cell development in mammals, whereas the paternal methylation is largely preserved in zebrafish (Ortega-Recalde et al., 2019; Skvortsova et al., 2019). In consist with previous findings, our observations in this study demonstrated that the family-specific differences in the genetic mechanism of sex determination can be attributed to the differences in epigenetic markers depending on inter-family genomic variation in this fish species. However, further experiments of interconnectedness of genetic component and epigenetic marks will enhance our understanding of genome function and induction of sexual phenotypic plasticity in this model animal.

Epigenetic marks can also be influenced by environmental factors (Kaminsky et al., 2009). Epigenetically regulated sex determination in response to the environment plays a key role in phenotypic plasticity. Species can modulate adaptive responses to environmental changes during evolution by retaining the epigenetic memory of environmental conditions experienced by their parents. Sex ratio variation mediated by epigenetic regulatory mechanisms in species with G × E may lead to maladaptive changes, resulting in a skewed sex ratio in the population (Le Galliard et al., 2005; Kronholm and Collins, 2015; Consuegra and Lopez, 2016). Taking into account that the sex ratio in domesticated zebrafish is family-specific (Liew et al., 2012; Ribas et al., 2017a), it could be influenced by epigenetic mechanisms, resulting in phenotypic sexual plasticity induced by environmental factors (Consuegra and Lopez, 2016; Valdivieso et al., 2020). Our study on DNA methylation in sex-biased zebrafish families revealed that the male-biased family exhibited strikingly higher number of methylated sites than the female-biased family in both testes and ovaries (Figure 4). Our further findings of the effect of epigenetic modifications within each sex-biased family demonstrated that testes exhibited considerably higher hypermethylated CpGs than the ovaries within both male-biased and female-biased families (Figure 6). Our observations suggest that the sex ratio variation in sex-biased zebrafish families with different genetic backgrounds of sex determination is influenced by epigenetic mechanisms.

Transgenerational inheritance of epigenetic modifications associated with differential DNA methylation regions (epimutations) is a plastic memory of organisms for phenotypic changes that can be transmitted to subsequent generations and cannot be explained by changes in primary DNA sequence and Mendelian genetics (Youngson and Whitelaw, 2008; Daxinger and Whitelaw, 2010, 2012; Shao et al., 2014; Skinner et al., 2014). Epigenetic inheritance in mammals revealed that the majority of environmentally induced epigenetic marks are erased and reset between generations (Daxinger and Whitelaw 2010, 2012; Feng et al., 2010). Unlike mammals, studies in zebrafish confirmed the heredity of the parental DNA methylome in the offspring through the sperm, which is not reset in early embryonic development (Macleod et al., 1999; Jiang et al., 2013; Potok et al., 2013). During the crucial time windows of embryonic development in zebrafish (50% epiboly to Prim-5, Kimmel et al., 1995; Abozaid et al., 2011; Hosseini et al., 2019a), the time windows that investigated in this study, the primordial germ cells could have been mostly affected by environmental temperature (Abozaid et al., 2011; Hosseini et al., 2019a) and would therefore be potentially more susceptible to establish novel environmentally induced DNA methylation patterns.

### Genes Associated With Genome-Wide DNA Methylation Changes

Epigenetic mechanisms such as DNA methylation can affect gene expression and regulate phenotypic sexual dimorphism in a sex-determining pathway (Lister et al., 2009; Feng et al., 2010; Zemach et al., 2010; Moore et al., 2013). Our results of chromosome-wise distribution of genes associated with DNA methylation changes revealed seven genes were common in all compared groups with a distinct family-biased methylation effect, including *viml, mvb12bb, pitpnb, rtn1a, sp8b, olfml2ba*, and *fbxl22*, located on chromosome 2, 8, 10, 13, 16, 20, and 25, respectively (Figure 5). The chromosomal distribution of these family-biased genes showed a biased methylation effect in testes and ovaries under both temperature conditions.

We also identified the family-specific genes (unique genes) within each sex-biased family comparing the testes and ovaries, where the male-biased family exhibited higher hypermethylated CpGs than the female-biased family, especially in the group exposed to high temperature (Figure 7). Therefore, we speculate that the family-related genes (family-biased and/or family-specific) may influence the activation of reproductive-related genes in downstream pathways to follow the trend of sex determination toward maleness or femaleness, thereby leading to a skewed sex ratio in the population. However, further experiments are required to validate our findings.

The phenotypic divergence between the two sexes requires sex-specific gene regulation and differential sex-biased gene expression because both sexes share the same genome (except for sex chromosomes), which can be influenced by epigenetic regulatory mechanisms (Ghiselli et al., 2012; Grath and Parsch, 2016). In agreement with previous evidence, our further findings also showed the effect of DNA methylation changes on candidate sex-related genes studied here in the zebrafish gonad (Figure 8), suggesting that the epigenetic modifications may influence sex-determining gene expression. Among the differentially methylated candidate reproductive genes, *fmr1* showed the highest number of associated DMCs in both families. This gene is involved in spermatogenesis and has a function in RNA regulation, which directly affects reproductive outcome by regulating the DNA damage response during spermatogenesis (Akemann et al., 2019). Other important genes involved in testicular differentiation and spermatogenesis with high methylated sites such as *lztfl1*, *sox9a*, *sox3*, and *gata4* were also identified in our study. For example, *sox9a* is a candidate pro-male gene that along with *dmrt1* and *amh* may have a function in Sertoli cell differentiation during zebrafish sex determination (Lee et al., 2017; Webster et al., 2017). Consistent with previous findings on the function of *sox9a* in zebrafish testes, we observed hypermethylation of this gene in testes versus ovaries, which may have an impact on the regulation of its expression. Likewise, the methylation of sex-biased genes involved in ovarian differentiation and folliculogenesis, such as *ctnnb1* and *lhx8a* (Lee et al., 2017; Hosseini et al., 2019b)*,* was also observed in our analysis. Notably, the methylation of the sex-biased genes showed that some of them, including *wt1a*, *prkcz*, *lhcgr*, *ints3*, and *gsdf*, were methylated only in the control group of both families, where for instance *wt1a* and *gsdf* have a known function in sex determination and gonad differentiation of male zebrafish (Lee et al., 2017). However, contrary to the control group, methylation of only one gene, *nr5a1a*, which has a function in steroidogenesis, was detected in the treatment group of both families. The same is true for candidate genes involved in epigenetic mechanisms, where *dnmt3bb.1* is identified only in the control group and *h1m* only in the treatment group. *Dnmt3bb.*1 is a DNA methyltransferase gene involved in the establishment of DNA methylation patterns during zebrafish gametogenesis (Campos et al., 2012; Akemann et al., 2019). However, the *linker histone h1m* gene is involved in encoding histone variants and is expressed in the primordial germ cells of the zebrafish gonad (Müller et al., 2002; Lee et al., 2017). Taken together, these results represent novel insights into the effect of DNA methylation changes on genes in zebrafish gonad in different sex-biased families, which can influence the phenotypic sexual dimorphism, resulting in imbalanced sex ratio in the population and provide a valuable resource of candidate genes for further research. The association between DNA methylation changes of the candidate genes studied here and gene expression requires further experiment.

### Methylation of Gene Promoters in Sex-Biased Families

Given that the DNA methylation of promoters as a functional regulatory element of the genome is highly related to gene expression (Lister et al., 2009; Feng et al., 2010; Zemach et al., 2010; Chen et al., 2017), we pursued the DNA methylation pattern in gene promoters of both sex-biased zebrafish families. Our results showed more hypermethylated promoters in the male-biased family than the female-biased family and a high number of DMPs in the testes compared with the ovaries (Supplementary Figures 6, 8). Studies of DNA methylation changes in sex-related gene promoters such as *cyp19a1a* in fish species showed different methylation patterns during folliculogenesis in olive flounder, *Paralichthys olivaceus* (Si et al., 2016) and zebrafish (Bai et al., 2016). Likewise, a study of DNA methylation changes in *cyp19a1a* promoter in European sea bass, *Dicentrarchus labrax,* results in suppression of its expression and male sexual development when incubated at high ambient temperature during early development (Navarro-Martin et al., 2011). In addition to single gene studies, research on genome-wide DNA methylation in fish species, such as the half-smooth tongue sole, revealed the effect of epigenetic regulatory mechanisms on the suppression of female-specific gene expression under high temperature, resulting in environmentally induced sex reversal (Shao et al., 2014). Global DNA methylation in Nile tilapia, *Oreochromis niloticus*, also represented an increase in the methylation levels of different chromosomes in both sexes in elevated temperature-exposed fish compared to the control group (Sun et al., 2016). A recent study on the genetic mechanisms underlying sex determination in European sea bass and its interaction with high ambient temperature revealed that temperature exposure leads to hypomethylation of *sox3* gene, resulting in up-regulation of its expression in both testes and ovaries (Geffroy et al., 2021). However, the DNA methylation level of *sox9a* was increased only in the testes, but no significant difference was observed in the ovaries. Interestingly, the standard association between the hypermethylation and down-regulation of gene expression was not detected for *sox9a* in the testes at high temperature condition (Geffroy et al., 2021). Altogether, previous studies have verified the effect of DNA methylation changes on promoters of sex-biased genes in the regulation of two different sexes and in the generation of environmentally induced sex reversal mediated by temperature. In this study, we reported unique evidence for DNA methylation changes of gene promoters in different sex-biased zebrafish families under two temperature conditions (Figure 9), which may regulate a family-biased sex ratio.

Our further investigation of candidate genes comparing the two sex-biased families showed that the promoters of some reproduction- and epigenetic-related genes, including *nabp1a*, *histh1l*, and *sox9b* were methylated only in the male-biased family, that were not detected in the female-biased family (Figure 10). Of these genes, hypermethylation of only the *histh1l*, a histone variant gene, was detected in the high incubation temperature group of the male-biased family, which may play a role in sexual development towards maleness. Consistent with our observation here, a previous study demonstrated the upregulation of *histh1l* in zebrafish testes compared to ovaries (Lee et al., 2017).

## Conclusion

In summary, we found more DNA methylation in the male-biased family than the female-biased family and a high number of methylated positions in the testes than the ovaries. The effect of ambient temperature also showed more methylation at the high incubation temperature than the control temperature. These observations lead us to conclude that the DNA methylation changes may influence sexual phenotypic plasticity as a response to the environmental conditions. Our study provides new insights into the epigenetic mechanisms underlying sex-biased zebrafish families and improves our understanding of sex ratio variation in species with G × E mechanism of sex determination in evolutionary developmental biology.

## Data Availability

The datasets presented in this study can be found in online repositories. The names of the repository/repositories and accession number(s) can be found here: https://osf.io/bax9y/. Further use of the datasets generated and analysed in this study requires the consent of the first corresponding author (SH).
