# Supplementary material for: Epigenetic Regulation of Phenotypic Sexual Plasticity Inducing Skewed Sex Ratio in Zebrafish
Source: Front Cell Dev Biol. 2022 Jul 15;10:880779. doi: 10.3389/fcell.2022.880779 (PMC9334531; doi:10.3389/fcell.2022.880779)
Supplement: Supplementary file 4 [file Table12.DOCX]

**Supplementary Table 12 |** The results of the functional analysis of significant gene ontology (GO) terms (p <0.05) for significantly differentially methylated gene promoters in testes versus ovaries of male-biased and female-biased family of zebrafish with the number of annotated genes and *P*-values

| GO term | Description | No. of genes | *P*-value |
| --- | --- | --- | --- |
| MCMB vs. FCMB |  |  |  |
| GO:0000122 | negative regulation of transcription by RNA polymerase II | 12 | 0.0003 |
| GO:0045892 | negative regulation of transcription, DNA-templated | 18 | 0.0070 |
| GO:0034401 | chromatin organization involved in regulation of transcription | 2 | 0.0347 |
| GO:0090090 | negative regulation of canonical Wnt signaling pathway | 3 | 0.0379 |
| MCFB vs. FCFB |  |  |  |
| GO:0030154 | cell differentiation | 54 | 0.0001 |
| GO:0006355 | regulation of transcription, DNA-templated | 44 | 0.0007 |
| GO:0000003 | reproduction | 6 | 0.0013 |
| GO:1902742 | apoptotic process involved in development | 2 | 0.0225 |
| MTMB vs. FTMB |  |  |  |
| GO:0031936 | negative regulation of chromatin silencing | 2 | 0.0084 |
| GO:0045910 | negative regulation of DNA recombination | 2 | 0.0224 |
| GO:0000187 | activation of MAPK activity | 2 | 0.0238 |
| GO:0060281 | regulation of oocyte development | 1 | 0.0455 |
| MTFB vs. FTFB |  |  |  |
| GO:0043516 | regulation of DNA damage response, signal transduction by p53 | 2 | 0.0041 |
| GO:0032918 | spermidine acetylation | 2 | 0.0041 |
| GO:0045944 | positive regulation of transcription by RNA polymerase II | 15 | 0.0076 |
| GO:2000050 | regulation of non-canonical Wnt signaling pathway | 2 | 0.0124 |

MCMB vs. FCMB (male control male-biased versus female control male-biased); MCFB vs. FCFB (male control female-biased versus female control female-biased); MTMB vs. FTMB (male treatment male-biased versus female treatment male-biased); MTFB vs. FTFB (male treatment female-biased versus female treatment female-biased).
