# Supplementary material for: Epigenetic Regulation of Phenotypic Sexual Plasticity Inducing Skewed Sex Ratio in Zebrafish
Source: Front Cell Dev Biol. 2022 Jul 15;10:880779. doi: 10.3389/fcell.2022.880779 (PMC9334531; doi:10.3389/fcell.2022.880779)
Supplement: Supplementary file 6 [file DataSheet1.pdf]

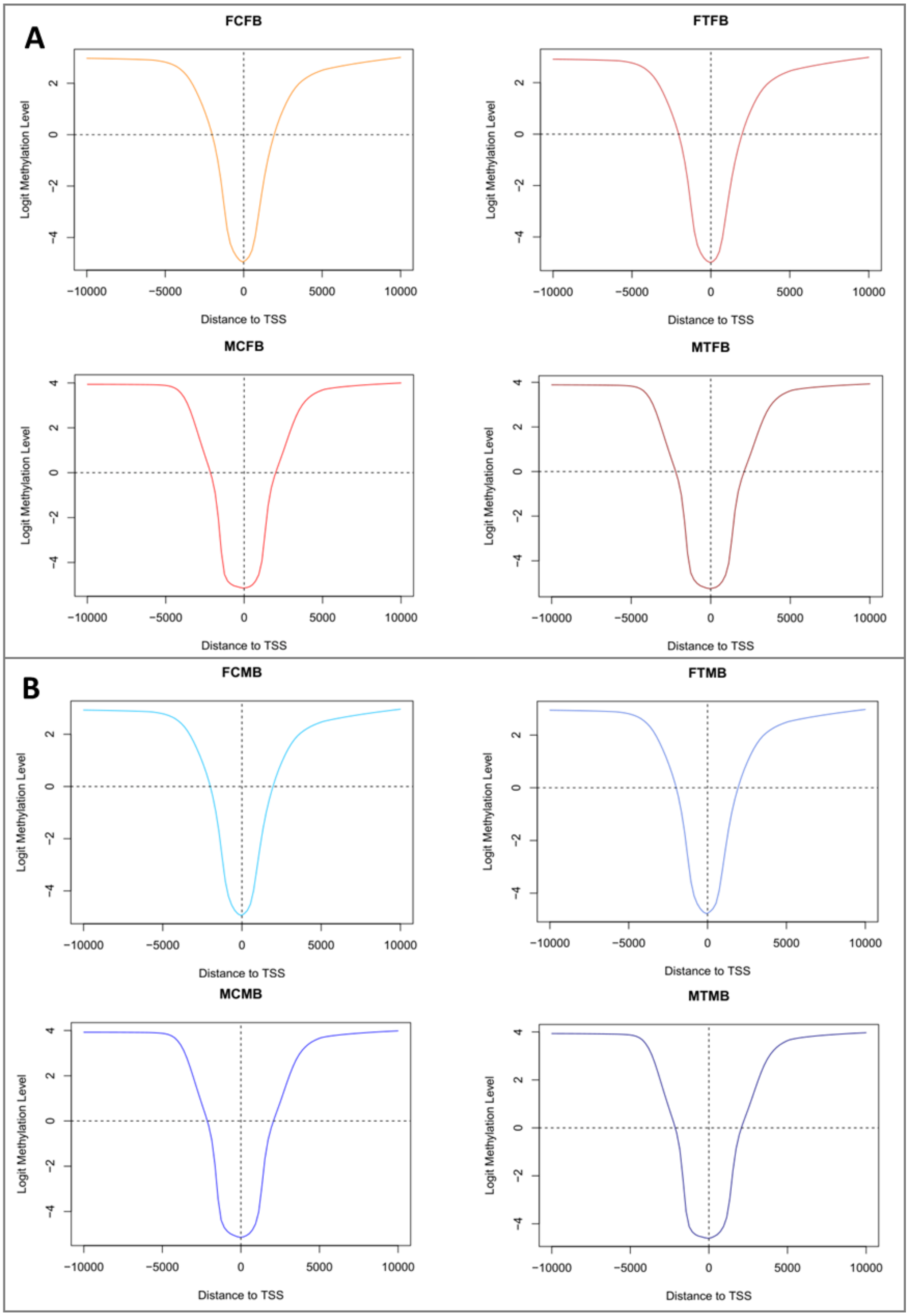

Supplementary Figure 1 | Distribution of DNA methylation around transcription start site (TSS). (**A**) FCFB (female control female-biased), FTFB (female treatment female-biased), MCFB (male control female-biased), MTFB (male treatment female-biased). (**B**) FCMB (female control male-biased), FTMB (female treatment male-biased), MCMB (male control male-biased), MTMB (male treatment male-biased).

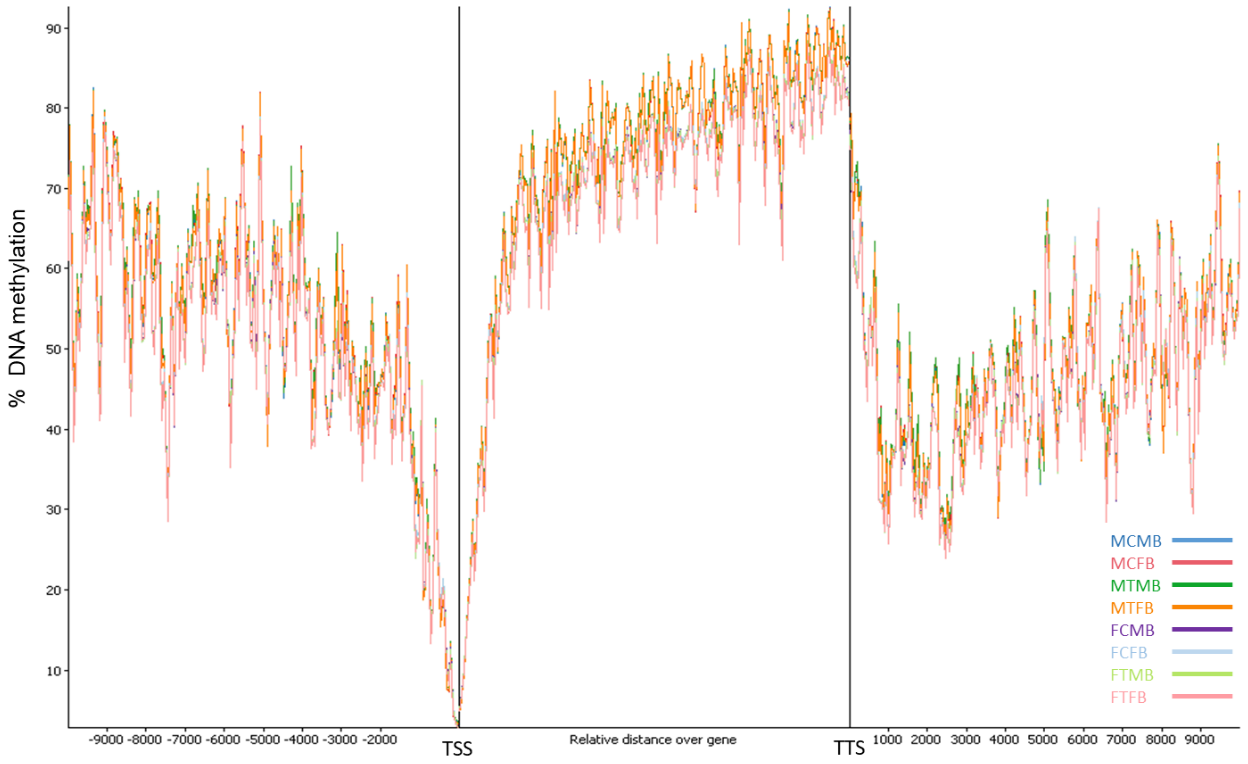

Supplementary Figure 2 | DNA methylation landscape across genes from up-stream transcription start site (TSS) to down-stream transcription termination site (TTS). MCMB (male control male-biased), MCFB (male control female-biased), MTMB (male treatment male-biased), MTFB (male treatment female-biased), FCMB (female control male-biased), FCFB (female control female-biased), FTMB (female treatment male-biased), FTFB (female treatment female-biased).

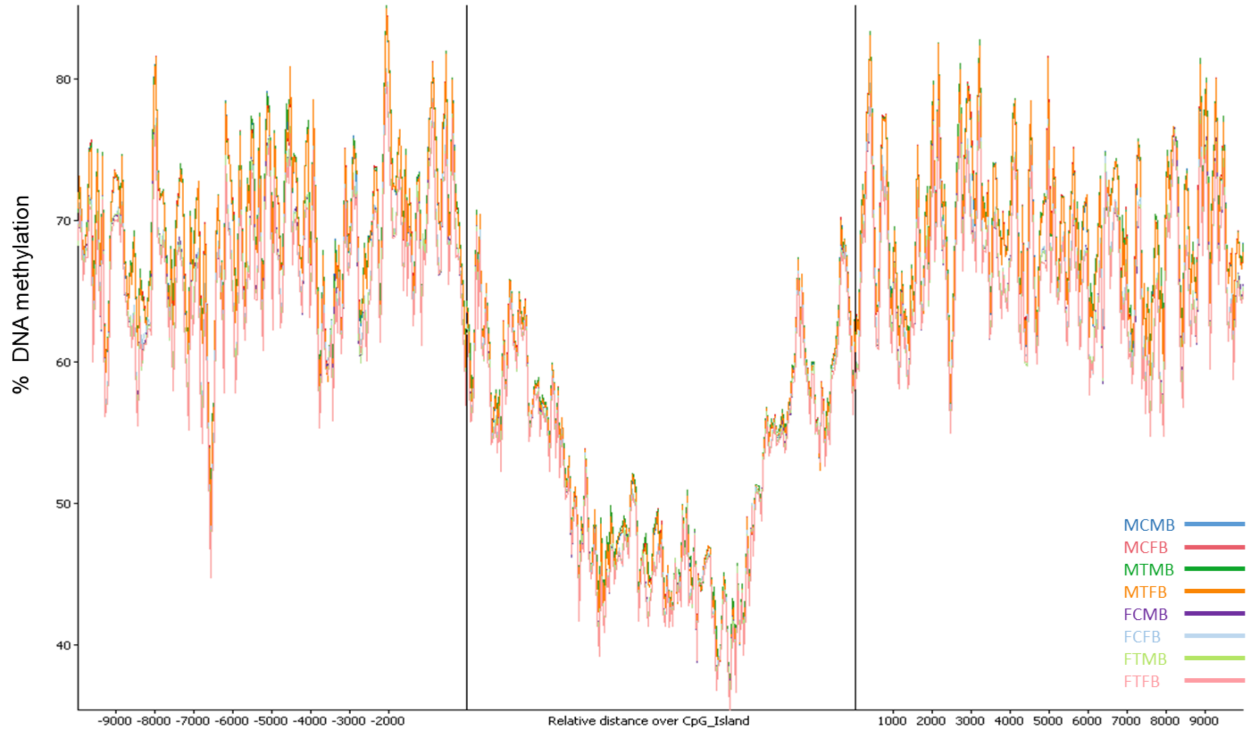

Supplementary Figure 3 | DNA methylation landscape over CpG islands. MCMB (male control male-biased), MCFB (male control female-biased), MTMB (male treatment male-biased), MTFB (male treatment female-biased), FCMB (female control male-biased), FCFB (female control female-biased), FTMB (female treatment male-biased), FTFB (female treatment female-biased).

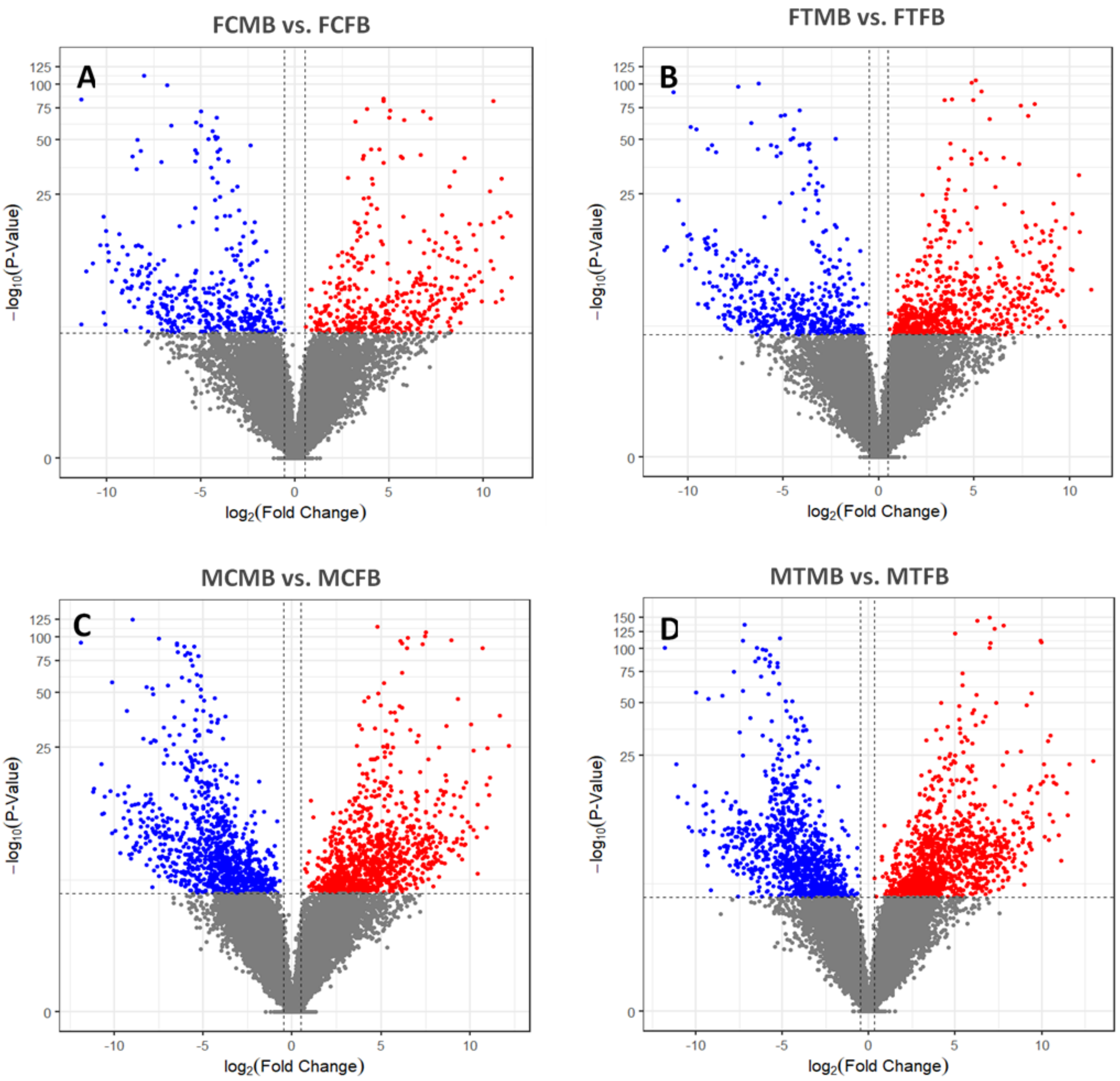

Supplementary Figure 4 | Differentially methylated CpG sites (DMCs) in zebrafish gonad of male-biased versus female-biased family. Volcano plots represent DMCs in ovaries in control (A) and treatment (B) groups of male-biased vs. female-biased family (FCMB vs. FCFB and FTMB vs. FTFB, respectively), and in testes in control (C) and treatment (D) groups of male-biased vs. female-biased family (MCMB vs. MCFB and MTMB vs. MTFB, respectively). The x-axis represents $\log_2$ (fold change) for each CpG site and the y-axis indicates $-\log_{10}$ (p-values). Red dots represent hypermethylated sites and blue dots represent hypomethylated sites. The significance methylation differences shown at threshold false discovery rate <0.05 (FDR <0.05).

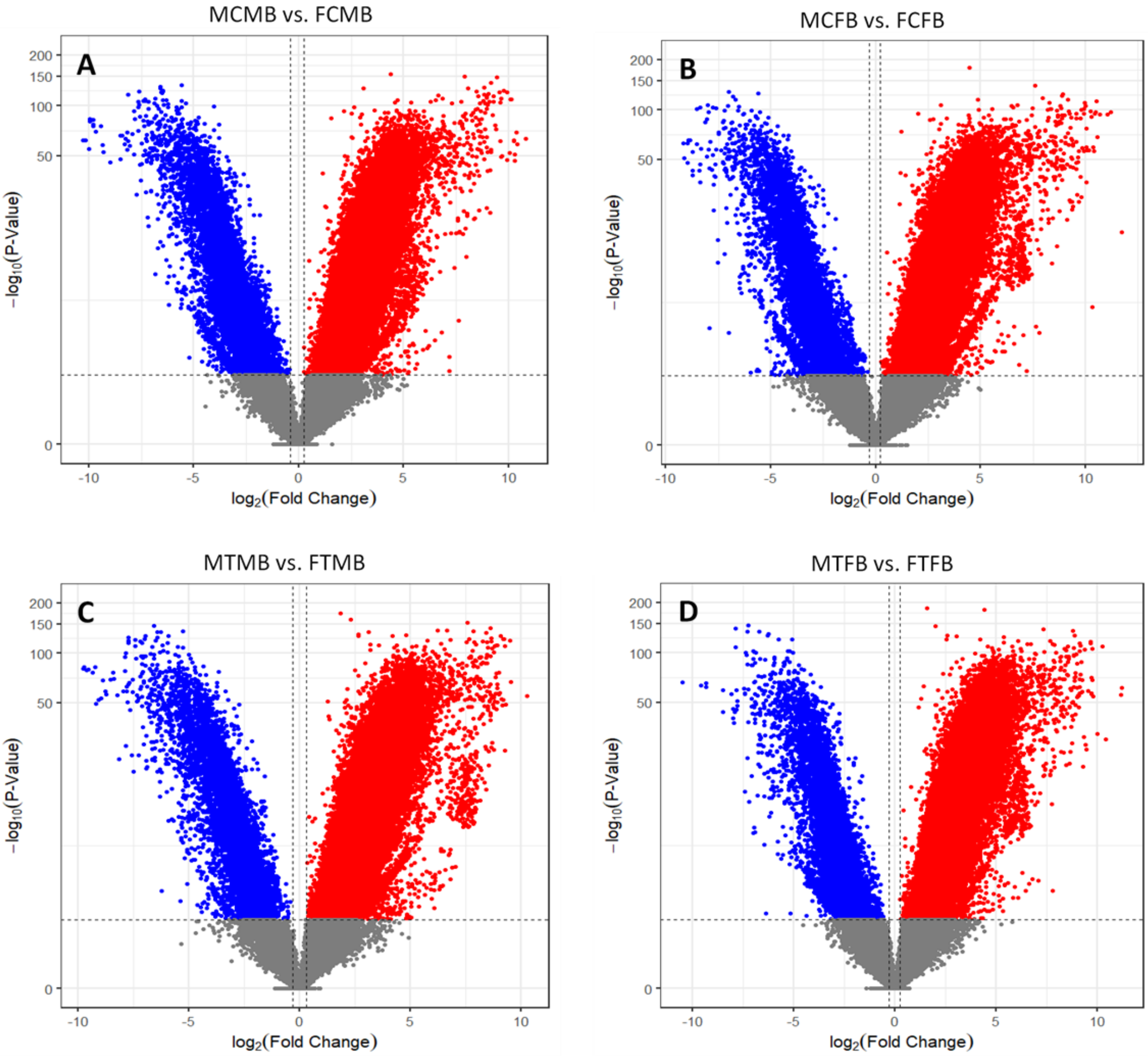

Supplementary Figure 5 | Differentially methylated CpG sites (DMCs) in testes versus ovaries within male-biased and female-biased zebrafish families. Volcano plots represent DMCs in testes vs. ovaries in control group of male-biased (A) and female-biased (B) families (MCMB vs. FCMB and MCFB vs. FCFB, respectively), and in testes vs. ovaries in treatment group of male-biased (C) and female-biased (D) families (MTMB vs. FTMB and MTFB vs. FTFB, respectively). The x-axis represents $\log_2$ (fold change) for each CpG site and the y-axis indicates $-\log_{10}$ (p-values). Red dots represent hypermethylated sites and blue dots represent hypomethylated sites. The significance methylation differences shown at threshold false discovery rate <0.05 (FDR <0.05).

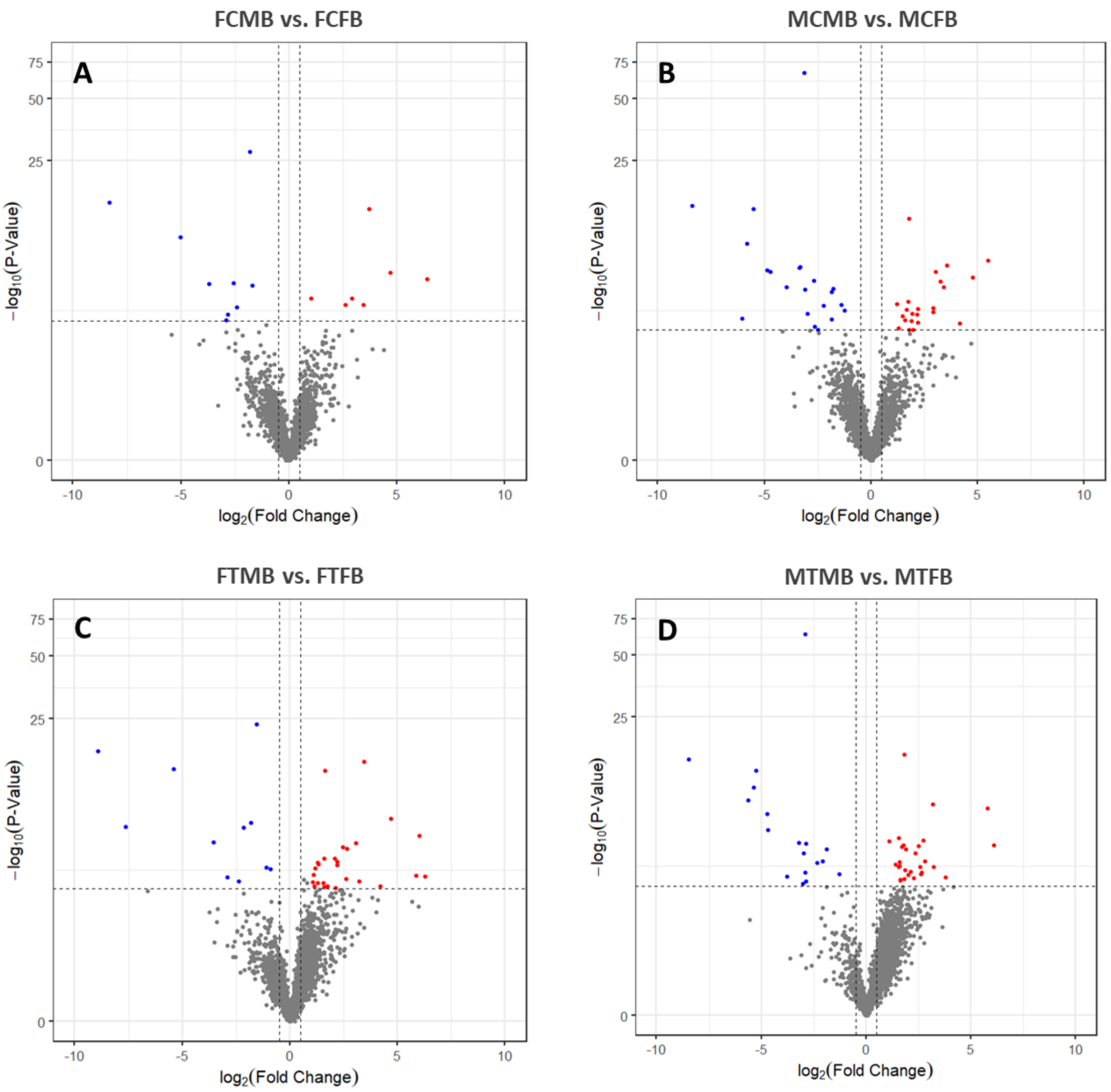

Supplementary Figure 6 | Differentially methylated promoters in male-biased versus female-biased family. (**A**) female control male-biased versus female control female-biased (FCMB vs. FCFB); (**B**) male control male-biased versus male control female-biased (MCMB vs. MCFB); (**C**) female treatment male-biased versus female treatment female-biased (FTMB vs. FTFB); (**D**) male treatment male-biased versus male treatment female-biased (MTMB vs. MTFB). The x-axis represents $\log_2$ (fold change) for each CpG site and the y-axis indicates $-\log_{10}$ (p-values). Red dots represent hypermethylated sites and blue dots represent hypomethylated sites. The significance methylation differences show at threshold false discovery rate <0.05 (FDR <0.05).

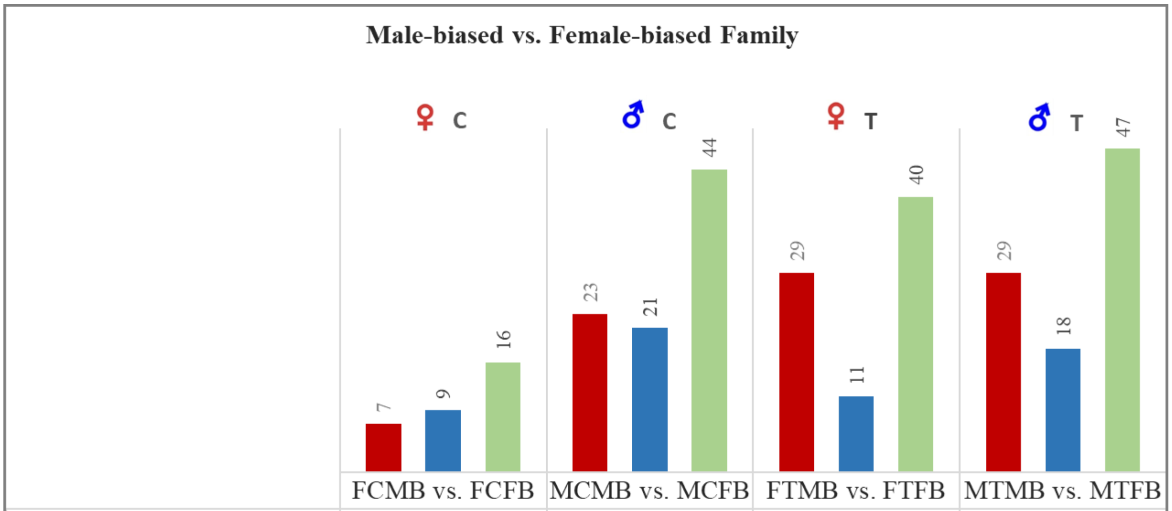

	FCMB vs. FCFB	MCMB vs. MCFB	FTMB vs. FTFB	MTMB vs. MTFB
Hypermethylated Promoters	7	23	29	29
Hypomethylated Promoters	9	21	11	18
Total	16	44	40	47

Supplementary Figure 7 | Overview of differentially methylated promoters in zebrafish gonad of male-biased versus female-biased family in control (C) and treatment (T) groups. female control male-biased versus female control female-biased (FCMB vs. FCFB); male control male-biased versus male control female-biased (MCMB vs. MCFB); female treatment male-biased versus female treatment female-biased (FTMB vs. FTFB); male treatment male-biased versus male treatment female-biased (MTMB vs. MTFB).

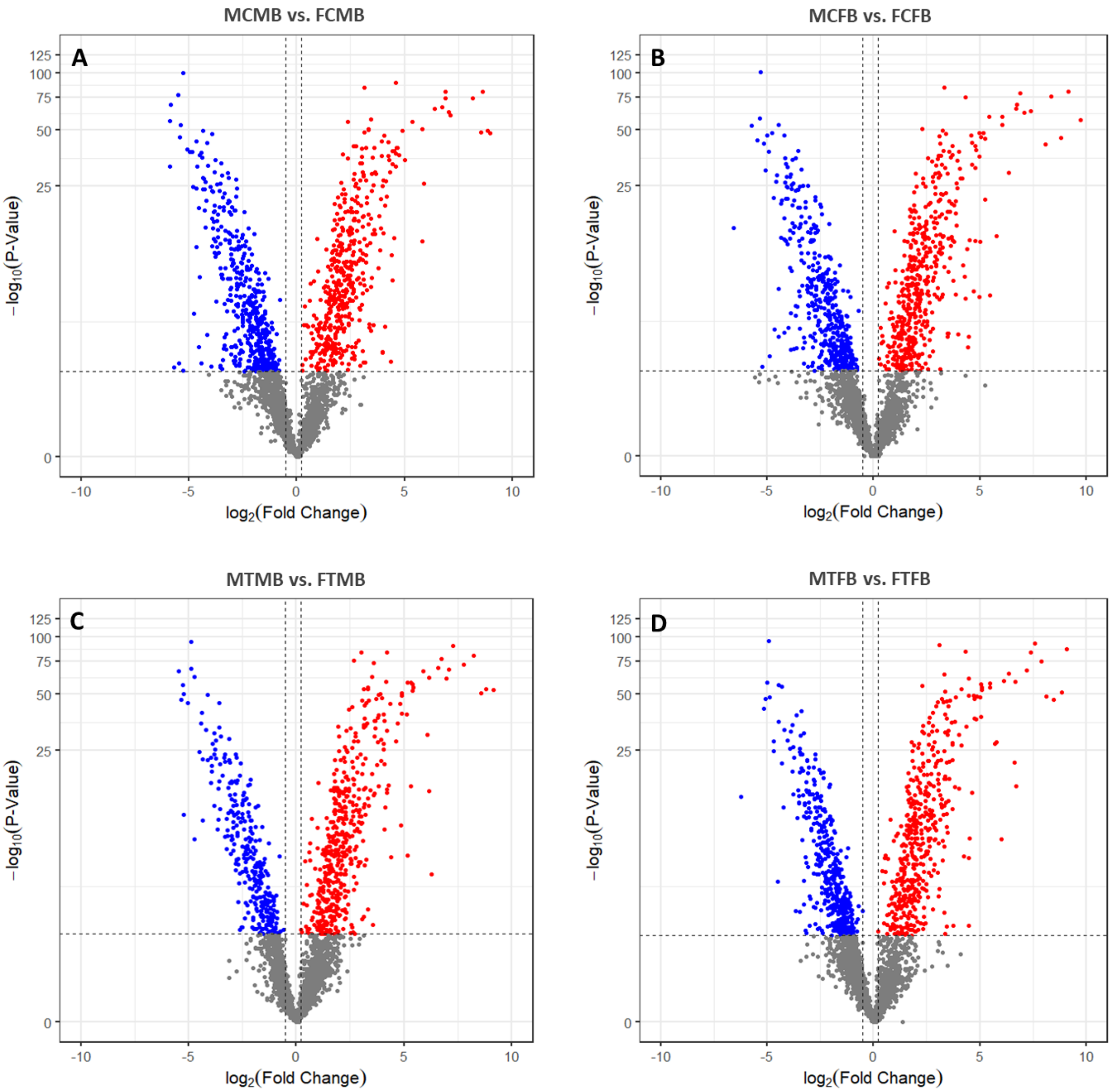

Supplementary Figure 8 | Differentially methylated promoters in testes versus ovaries within male-biased and female-biased families. (**A**) male control male-biased versus female control male-biased (MCMB vs. FCMB); (**B**) male control female-biased versus female control female-biased (MCFB vs. FCFB); (**C**) male treatment male-biased versus female treatment male-biased (MTMB vs. FTMB); (**D**) male treatment female-biased versus female treatment female-biased (MTFB vs. FTFB). The x-axis represents $\log_2$ (fold change) for each CpG site and the y-axis indicates $-\log_{10}$ (p-values). Red dots represent hypermethylated sites and blue dots represent hypomethylated sites. The significance methylation differences show at threshold false discovery rate <0.05 (FDR <0.05).

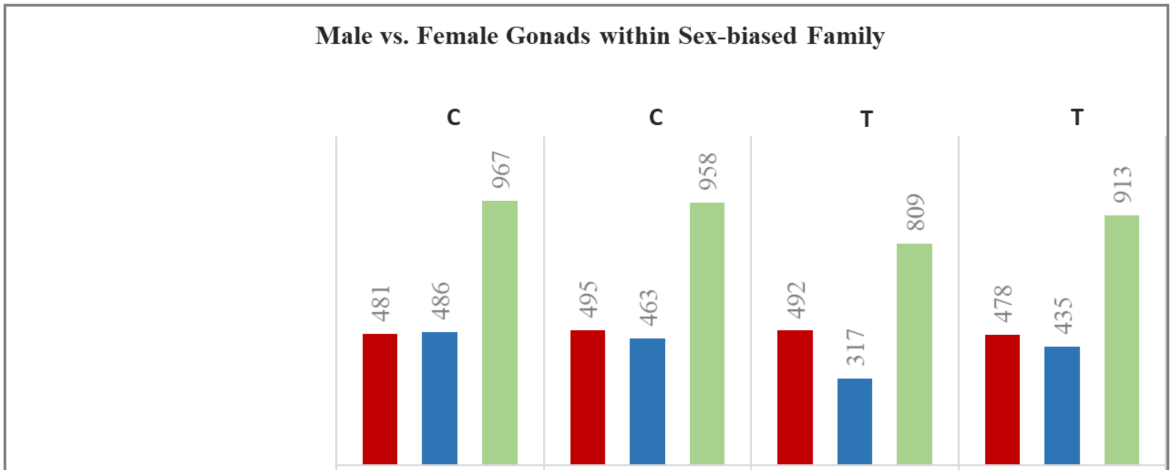

	MCMB vs. FCMB	MCFB vs. FCFB	MTMB vs. FTMB	MTFB vs. FTFB
Hypermethylated Promoters	481	495	492	478
Hypomethylated Promoters	486	463	317	435
Total	967	958	809	913

Supplementary Figure 9 | Overview of differentially methylated promoters in testes versus ovaries within male-biased and female-biased family in control (C) and treatment (T) groups. male control male-biased versus female control male-biased (MCMB vs. FCMB); male control female-biased versus female control female-biased (MCFB vs. FCFB); male treatment male-biased versus female treatment male-biased (MTMB vs. FTMB); male treatment female-biased versus female treatment female-biased (MTFB vs. FTFB).
