# Supplementary material for: Epigenetic Regulation of Phenotypic Sexual Plasticity Inducing Skewed Sex Ratio in Zebrafish
Source: Front Cell Dev Biol. 2022 Jul 15;10:880779. doi: 10.3389/fcell.2022.880779 (PMC9334531; doi:10.3389/fcell.2022.880779)
Supplement: Supplementary file 9 [file Table13.DOCX]

**Supplementary Table 13 |** The results of the functional analysis of significant pathways (p <0.05) for significantly differentially methylated gene promoters in testes versus ovaries of male-biased and female-biased family of zebrafish with the number of annotated genes and *P*-values

| Pathway | No. of genes | *P*-value |
| --- | --- | --- |
| MCMB vs. FCMB |  |  |
| Apoptosis | 4 | 0.0235 |
| MAPK6/MAPK4 signaling | 2 | 0.0381 |
| Stabilization of p53 | 2 | 0.0381 |
| TNFR2 non-canonical NF-kB pathway | 2 | 0.0381 |
| MCFB vs. FCFB |  |  |
| Signaling by Wnt | 5 | 0.0393 |
| MTMB vs. FTMB |  |  |
| DAP12 interactions | 2 | 0.0213 |
| Downstream signal transduction | 2 | 0.0213 |
| Signaling by EGFR | 2 | 0.0213 |
| Immune System | 5 | 0.0275 |
| MTFB vs. FTFB |  |  |
| Processing of Capped Intron-Containing Pre-mRNA | 4 | 0.0405 |

MCMB vs. FCMB (male control male-biased versus female control male-biased); MCFB vs. FCFB (male control female-biased versus female control female-biased); MTMB vs. FTMB (male treatment male-biased versus female treatment male-biased); MTFB vs. FTFB (male treatment female-biased versus female treatment female-biased).
